# Association of lipoprotein(a) and composite inflammatory indices with functional outcomes in acute ischemic stroke: a prospective cohort study

**DOI:** 10.1186/s12944-026-02975-6

**Published:** 2026-05-11

**Authors:** Yi Tang, Yueyu Zhang, Xinyi Chen, Zilong Yue, Yu Wang, Shuaizhou Wang, Xun He, Yuankui Wu, Jie Hu, Zhonghua Sun, Juncang Wu

**Affiliations:** 1 Department of Neurology, Anhui Medical University, Hefei, 230032 China; 2 Department of Neurology, The Second People’s Hospital of Hefei, Hefei, 230011 China; 3 Department of General Surgery, Guoyang Branch of Anhui Provincial Hospital, Bozhou, 233500 China; 4 Medical School of Nanjing University, Nanjing, 210093 China

**Keywords:** Acute ischemic stroke, Lipoprotein(a), Inflammation-related indices, C-reactive protein-to-albumin ratio, Functional outcome

## Abstract

**Background:**

Dysregulated lipid metabolism and systemic inflammatory cascades significantly disrupt the neurofunctional trajectory of acute ischemic stroke (AIS). However, the precise pathomechanisms underlying the interactive pathogenic effects of lipoprotein(a) [Lp(a)] and whole-blood composite inflammatory markers in prognostic interventions remain elusive. This study aims to investigate whether Lp(a) and multiple traditional blood inflammation markers jointly influence the early and 90-day functional outcomes in elderly AIS patients.

**Methods:**

This prospective cohort study enrolled patients aged ≥ 60 years diagnosed with AIS. Baseline Lp(a) levels and seven systemic inflammatory indices, including the C-reactive protein-to-albumin ratio (CAR), were measured upon admission. The primary endpoint was poor functional outcome, defined as a modified Rankin Scale (mRS) score ≥ 3 at 90 days post-onset. The associations were evaluated using multivariable logistic regression, weighted quantile sum (WQS) regression, and exploratory mediation analyses.

**Results:**

Among 732 included patients, 108 (14.8%) experienced a poor functional outcome at 90 days. In fully adjusted models, each 1-standard deviation (SD) increase in Lp(a) was independently associated with a more than twofold higher odds of a 90-day poor outcome (odds ratio [OR], 2.12; 95% CI, 1.70–2.66). Patients in the highest Lp(a) tertile faced significantly increased risk compared with those in the lowest tertile (OR, 2.74; 95% CI, 1.52–5.07). Among the evaluated inflammatory indices, CAR showed the most robust independent association with 90-day poor outcomes (OR per 1-SD increase, 1.59; 95% CI, 1.28–2.13). Notably, patients presenting with both high Lp(a) and high CAR exhibited the greatest risk for an adverse 90-day prognosis (OR, 4.60; 95% CI, 2.29–9.81). Compared with individual inflammatory markers, Lp(a) demonstrated modest prognostic discriminatory ability (AUC, 0.696). Exploratory mediation analysis suggested that CAR mediated only a limited proportion of the association between Lp(a) and 90-day poor outcome, with an indirect effect estimate of 0.008 (95% CI, 0.002–0.026; *P* = 0.011).

**Conclusions:**

Elevated baseline Lp(a) and CAR were independently associated with poor early and 90-day functional outcomes in this cohort of older patients with AIS. Concurrent elevation of Lp(a) and CAR was associated with the highest odds of adverse outcome.

**Supplementary Information:**

The online version contains supplementary material available at 10.1186/s12944-026-02975-6.

## Background

 Acute ischemic stroke (AIS) remains a leading cause of long-term disability [1]. According to recent global estimates, stroke resulted in 11.9 million new events, affected 93.8 million people living with the condition, caused 7.3 million deaths, and contributed to 160.5 million disability-adjusted life-years in 2021. Among these cases, ischemic stroke represented roughly two-thirds (65.3%) of incident strokes worldwide [2]. While acute revascularization strategies salvage ischemic tissue, the trajectory of post-stroke functional recovery is largely governed by secondary biological cascades, particularly the interplay between atherogenic lipid pathways and systemic inflammation [3, 4].Following cerebral ischemia, the disruption of the blood-brain barrier and subsequent neurovascular inflammation profoundly influence infarct evolution and neurological outcomes [5, 6]. Consequently, circulating inflammatory and metabolic biomarkers are increasingly recognized not merely as bystanders, but as active modulators of stroke prognosis that could inform early risk stratification beyond conventional clinical metrics [7].

Among these pathophysiological drivers, lipoprotein(a) [Lp(a)]—a genetically determined, low-density lipoprotein-like particle—exhibits highly proatherogenic, prothrombotic, and proinflammatory properties [8]. High circulating Lp(a) levels carry oxidized phospholipids that directly promote endothelial injury and vascular inflammation [9].

Accumulating epidemiological research indicates that lipoprotein(a) plays a causal role in the development of atherosclerotic cardiovascular disease [10]; however, its value in predicting early functional outcomes after acute ischemic stroke remains insufficiently understood. Furthermore, the pathogenic potential of Lp(a) is hypothesized to be amplified by a systemic inflammatory environment. Yet, evidence derived primarily from coronary artery disease cohorts yielded conflicting results, with prior investigations reporting both synergistic and strictly independent effects of Lp(a) and conventional inflammatory markers such as high-sensitivity C-reactive protein [11–14]. Moreover, these studies were conducted mainly in coronary artery disease or acute myocardial infarction populations and relied largely on conventional inflammatory markers such as high-sensitivity C-reactive protein. Whether a similar interplay exists between Lp(a) and broader inflammatory burden in relation to post-stroke functional recovery remains unclear.

Crucially, existing literature largely evaluates individual inflammatory markers in isolation, which fails to comprehensively capture the multidimensional nature of the post-ischemic systemic response [5, 15]. Composite inflammatory biomarkers derived from routine laboratory tests—including the systemic inflammation response index (SIRI) [16], the ratio of neutrophils to lymphocytes (NLR) [17], and the ratio between C-reactive protein and albumin (CAR)—reflect interactions between immune activation and metabolic stress, and may therefore provide improved prognostic information while remaining easily obtainable in clinical practice [18, 19]. However, whether a high global inflammatory burden modifies the clinical impact of Lp(a) in the acute stroke setting, or whether specific inflammatory axes mediate the detrimental effects of Lp(a) on neurological recovery, is currently unknown.

To address these critical knowledge gaps, we prospectively evaluated the prognostic utility of Lp(a) alongside a comprehensive panel of seven inflammation-related composite indices (SIRI, NLR, MLR, dNLR, CAR, MHR, and GLR) in a cohort of older patients with AIS. The primary objective was to assess associations with poor functional outcome at 90 days, with 14-day poor functional outcome analyzed as a prespecified secondary endpoint reflecting early functional status. Beyond evaluating individual associations, we applied weighted quantile sum (WQS) regression to assess the combined mixture effects of these biomarkers. Additionally, exploratory mediation analysis allowing for exposure–mediator interaction was conducted to examine whether CAR might statistically account for part of the association between Lp(a) and post-stroke functional recovery.

## Methods

### Study population

This prospective cohort study consecutively enrolled patients with acute ischemic stroke (AIS) who were admitted to the Department of Neurology, Hefei Hospital Affiliated to Anhui Medical University, from March 2023 to December 2025. Consecutive enrollment was applied to minimize selection bias and to ensure that the study population was representative of routine clinical practice. The diagnosis of AIS was established according to the World Health Organization criteria and confirmed by cranial computed tomography (CT) and/or magnetic resonance imaging (MRI) [20]. Baseline demographic characteristics, clinical information, laboratory parameters, and neuroimaging findings were collected after admission using a standardized protocol. Overall, 798 patients were screened during the study period, and those meeting the eligibility criteria were included in the final analysis.

Patients were included if they had imaging-confirmed AIS, were aged ≥ 60 years, and had complete baseline data. Exclusion criteria comprised presentation > 72 h after onset; alternative cerebrovascular diagnoses (e.g., cerebral venous thrombosis or other non-index vascular ischemia); intracranial tumors, malignancy-related stroke, or intracranial infection; major non-neurological conditions accounting for neurological dysfunction (including active malignancy, severe infection, hematologic disease, advanced heart/hepatic/renal failure, or bleeding tendency); prior stroke with ongoing antiplatelet/anticoagulant therapy; reperfusion treatment for the index event (intravenous thrombolysis and/or endovascular thrombectomy, *n* = 22), to reduce treatment-related heterogeneity; ischemic stroke within the previous 3 months (*n* = 18); inability to cooperate due to dementia or psychiatric disorders (*n* = 16); chronic inflammatory disease; or missing key clinical/laboratory variables.

**Fig. 1:**
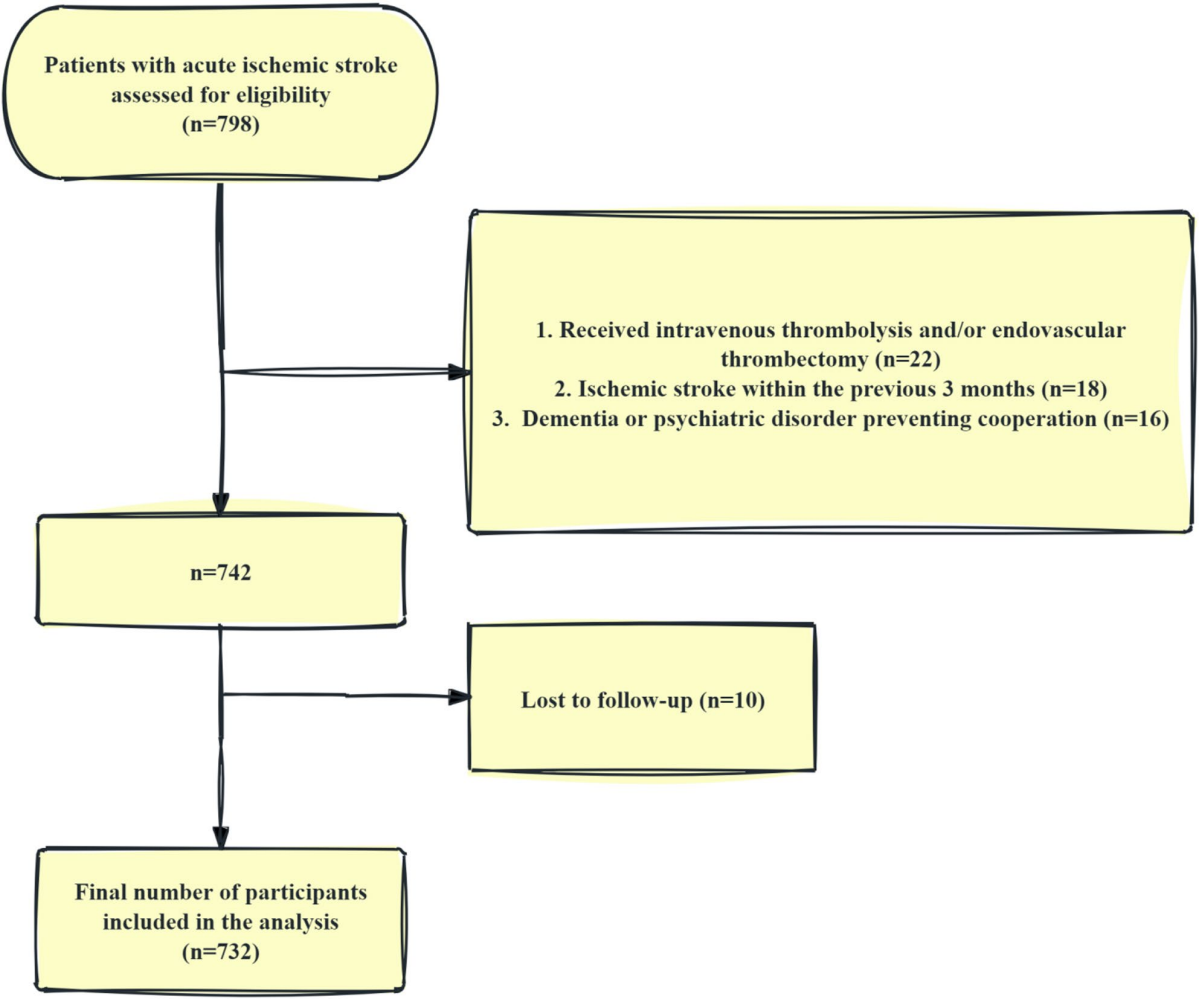
Flow chart of participants’ selection

After screening, 742 participants were enrolled at baseline. Data collection was completed at 90 days through outpatient follow-up or electronic medical records; 10 patients were lost to follow-up, resulting in 732 participants included in the primary analysis (Fig. 1).

### Outcomes and exposure factors

Functional status was evaluated using the modified Rankin Scale (mRS) by trained assessors who were blinded to baseline laboratory data. The primary outcome of this study was poor functional outcome at 90 days after stroke onset. A prespecified secondary outcome was poor functional outcome at 14 days after the event, which was selected to capture early post-stroke functional status during a clinically meaningful early post-stroke window and to approximate discharge-stage functional status in our clinical setting, rather than stable long-term recovery [21]. Poor outcome was defined a priori as an mRS score ≥ 3 at the corresponding time point.

Baseline blood samples were collected the following morning under standardized conditions (within 24 h of admission), requiring fasting at the time of collection. Lipoprotein(a) [Lp(a)] measurements were reported in mg/dL. Additionally, based on routine laboratory parameters collected at baseline, a series of inflammation-related composite indices were calculated, including: Systemic Inflammatory Response Index, Neutrophil-to-Lymphocyte Ratio, Monocyte-to-Lymphocyte Ratio, Derived Neutrophil-to-Lymphocyte Ratio, C-Reactive Protein-to-Albumin Ratio, Monocyte-to-High-Density Lipoprotein Ratio, and Glucose-to-Lymphocyte Ratio. These indices were calculated using preset formulas based on absolute blood cell counts and biochemical measurements; detailed calculation formulas are provided in the supplementary materials.

### Covariates

To enhance the robustness of the association between exposure variables and functional outcomes, we prespecified a set of clinically relevant covariates based on prior literature and biological plausibility. These covariates included age, sex, body mass index (BMI), smoking status, alcohol consumption, physical activity status, hypertension, diabetes mellitus, use of antihypertensive drugs, use of antidiabetic drugs, heart disease, stroke etiology (TOAST classification), and baseline stroke severity as assessed by the admission National Institutes of Health Stroke Scale (NIHSS). Smoking status was categorized as “yes” or “no” according to current smoking at admission (as documented in the medical record). Alcohol consumption was similarly dichotomized based on current drinking status. Physical activity status was classified as “active” or “inactive” using the predefined criteria applied in our case report form and routine admission assessment. Hypertension, diabetes mellitus, heart disease, use of antihypertensive drugs, and use of antidiabetic drug were defined by a prior physician diagnosis and/or documented use of disease-specific medications before admission, supplemented by in-hospital diagnostic confirmation when applicable. Ischemic stroke subtype was determined according to the Trial of Org 10,172 in Acute Stroke Treatment (TOAST) etiologic classification and grouped as large-artery atherosclerosis (LAA), cardioembolism (CE), or small-vessel occlusion (SVO). Baseline laboratory parameters collected at admission were recorded as continuous variables, including neutrophil count (NC), lymphocyte count (LC), monocyte count (MONO), white blood cell count (WBC), C-reactive protein (CRP), albumin (Alb), high-density lipoprotein cholesterol (HDL-C), and fasting blood glucose (FBG), with units consistent with the hospital laboratory reporting system. These variables were used either as adjustment covariates and/or as components for constructing composite inflammatory indices.

### Statistical analysis

Baseline characteristics are reported as mean (SD) or median (interquartile range) for continuous variables and as n (%) for categorical variables; groups were compared using Student’s t test or the Mann–Whitney U test for continuous variables and χ² or Fisher’s exact tests for categorical variables. Associations of Lp(a), SIRI, NLR, MLR, dNLR, CAR, MHR, and GLR with poor functional outcome at 14 and 90 days were evaluated using logistic regression, with effect estimates expressed as odds ratios (ORs) and 95% confidence intervals (CIs). Exposures were modelled primarily per 1-SD increase, whereas tertile-based analyses were retained as complementary analyses to facilitate comparison across exposure categories and to improve interpretability of extreme-group contrasts. We fitted a crude model, Model 1 adjusting for age, sex, BMI, smoking, alcohol use, and physical activity, and Model 2 further adjusting for hypertension, diabetes mellitus, heart disease, use of antihypertensive and antidiabetic drugs, TOAST subtype, and admission NIHSS. Predictive performance and clinical utility for both outcomes were assessed using ROC/AUC and decision curve analysis (DCA), and incremental predictive value was quantified using continuous NRI and IDI with bootstrap CIs. The multidimensional analytical framework used in this study was informed in part by our group’s previous methodological applications of multi-indicator models [22–24]. These references are cited here as methodological precedents rather than as direct evidence for the present exposure–outcome associations. Similar multivariable and multi-index analytical approaches have also been reported in externally published studies [25, 26]. For the 90-day outcome, weighted quantile sum (WQS) regression was used to evaluate the joint effect of the marker mixture and estimate component weights. To assess the combined association of inflammation and Lp(a) with outcomes, participants were cross-classified into four groups according to CAR (dichotomized at the median) and Lp(a) (dichotomized at 30 mg/dL, a commonly used clinical threshold [27]), and group-specific odds ratios for poor outcomes at 14 and 90 days were estimated using multivariable logistic regression. For the 90-day outcome, subgroup analyses were performed by age, sex, and smoking status. In addition, an exploratory counterfactual-based mediation analysis with an exposure–mediator interaction (Lp(a)×CAR) was conducted to examine whether CAR might statistically account for part of the association between Lp(a) and poor functional outcome. The Lp(a) contrast was defined as P75 versus P25, and ACME/ADE estimates were obtained using 2,000 bootstrap samples under full adjustment. Because Lp(a) and CAR were measured concurrently at baseline, this analysis was intended to provide an exploratory statistical decomposition rather than to establish a definitive temporally ordered causal pathway.

The primary analysis was conducted in a predefined cohort (*n* = 732) that excluded patients who received reperfusion therapy. The sensitivity analysis consisted of four components: (1) multiple comparison correction using the false discovery rate (FDR); (2) E-value tests for potential unmeasured confounders; and (3) inverse probability of treatment weighting (IPTW); (4) re-inclusion of the 22 patients who received reperfusion therapy for the primary event and additional adjustment for reperfusion therapy status in the fully adjusted logistic regression model.

All analyses were performed using R software (version 4.3.0); unless otherwise specified, two-sided *P *values < 0.05 were considered statistically significant. All primary clinical interpretations were centered on the 90-day outcome, whereas analyses of the 14-day outcome were considered secondary and supportive, intended to reflect early functional status rather than definitive recovery.

## Results

### Baseline characteristics

Among the 732 patients included in the final analysis, 108 (14.8%) had poor functional outcomes at 90 days, whereas 624 (85.2%) achieved good outcomes. In addition, poor functional status at 14 days was markedly more frequent among patients who subsequently remained in the poor-prognosis group at 90 days (87% vs. 23%, *P* < 0.001), suggesting substantial continuity between early functional status and later outcome.

With regard to baseline demographic and clinical characteristics, patients with poor outcomes were significantly older than those with good outcomes (75.36 ± 9.42 vs. 69.30 ± 9.20 years, *P* < 0.001) and had lower body mass index (23.35 ± 3.63 vs. 24.47 ± 3.43 kg/m², *P* = 0.003). Female sex was more common in the poor-outcome group (47% vs. 37%), whereas current smoking was less frequent (19% vs. 33%; both *P* < 0.05). The prevalence of heart disease was higher among patients with poor outcomes (16% vs. 8.8%, *P* = 0.026), and the use of antidiabetic drugs was also more frequent in this group (33% vs. 24%, *P* = 0.037). No significant between-group differences were observed for alcohol consumption, physical activity, hypertension, diabetes mellitus, or antihypertensive drug use.

Stroke etiology differed significantly between groups (*P* = 0.014). Cardioembolism was more common among patients with poor outcomes (32% vs. 22%), whereas small-vessel occlusion was less frequent (20% vs. 32%); the proportion of large-artery atherosclerosis was similar between groups. Stroke severity at admission was significantly higher among patients with poor outcomes, as indicated by elevated NIHSS scores compared with those with good outcomes (7.74 ± 5.69 vs. 3.39 ± 2.76, *P* < 0.001).

Patients experiencing poor functional outcomes demonstrated lower circulating lymphocyte counts together with markedly elevated CRP levels compared with those with good outcomes (1.54 ± 0.58 vs. 1.78 ± 2.45 × 10⁹/L, *P* = 0.034; 18.50 ± 37.27 vs. 5.09 ± 13.23 mg/L, *P* < 0.001). By contrast, neutrophil count, monocyte count, white blood cell count, albumin, HDL-C, and fasting blood glucose did not differ significantly between groups. Among the exposure biomarkers of primary interest, both Lp(a) (36.32 ± 26.89 vs. 22.27 ± 17.75 mg/dL, *P* < 0.001) and CAR (0.50 ± 1.05 vs. 0.13 ± 0.35, *P* < 0.001) were significantly elevated in patients with poor outcomes. In contrast, the other inflammation-related indices, including SIRI, NLR, MLR, dNLR, MHR, and GLR, showed no statistically significant between-group differences (all *P* > 0.05) (Table 1).

**Table 1:** Baseline characteristics of the study population

Characteristics	Total (*N* = 732)	Good Prognosis, *N* = 624	Poor Prognosis, *N* = 108	*p*-value
14-day outcome (OUT), *n* (%)				< 0.001
Good outcome (14-day)	493 (67%)	479 (77%)	14 (13%)	
Poor outcome (14-day)	239 (33%)	145 (23%)	94 (87%)	
Age, years	70.20 ± 9.47	69.30 ± 9.20	75.36 ± 9.42	< 0.001
Sex, *n* (%)				0.048
Male	449 (61%)	392 (63%)	57 (53%)	
Female	283 (39%)	232 (37%)	51 (47%)	
Body mass index (BMI), kg/m²	24.30 ± 3.48	24.47 ± 3.43	23.35 ± 3.63	0.003
Smoking status, *n* (%)				0.002
No	505 (69%)	417 (67%)	88 (81%)	
Yes	227 (31%)	207 (33%)	20 (19%)	
Alcohol consumption, *n* (%)				0.219
No	541 (74%)	456 (73%)	85 (79%)	
Yes	191 (26%)	168 (27%)	23 (21%)	
Physical activity status, *n* (%)				0.737
Inactive	343 (47%)	294 (47%)	49 (45%)	
Active	389 (53%)	330 (53%)	59 (55%)	
Hypertension, *n* (%)				0.758
No	233 (32%)	200 (32%)	33 (31%)	
Yes	499 (68%)	424 (68%)	75 (69%)	
Diabetes mellitus, *n* (%)				0.270
No	527 (72%)	454 (73%)	73 (68%)	
Yes	205 (28%)	170 (27%)	35 (32%)	
Heart disease, *n* (%)				0.026
No	660 (90%)	569 (91%)	91 (84%)	
Yes	72 (9.8%)	55 (8.8%)	17 (16%)	
TOAST etiologic classification (TOAST), *n* (%)				0.014
Large-artery atherosclerosis (LAA)	337 (46%)	286 (46%)	51 (47%)	
Cardioembolism (CE)	172 (23%)	137 (22%)	35 (32%)	
Small-vessel occlusion (SVO)	223 (30%)	201 (32%)	22 (20%)	
Antihypertensive drug, *n* (%)				0.695
No	263 (36%)	226 (36%)	37 (34%)	
Yes	469 (64%)	398 (64%)	71 (66%)	
Antidiabetic drugs, *n* (%)				0.037
No	547 (75%)	475 (76%)	72 (67%)	
Yes	185 (25%)	149 (24%)	36 (33%)	
Admission National Institutes of Health Stroke Scale score (NIHSS), points	4.03 ± 3.69	3.39 ± 2.76	7.74 ± 5.69	< 0.001
Neutrophil count (NC), ×10^9/L	5.14 ± 9.08	5.17 ± 9.80	4.92 ± 2.01	0.569
Lymphocyte count (LC), ×10^9/L	1.74 ± 2.27	1.78 ± 2.45	1.54 ± 0.58	0.034
Monocyte count (MONO), ×10^9/L	0.47 ± 0.53	0.47 ± 0.57	0.45 ± 0.19	0.458
White blood cell count (WBC), ×10^9/L	6.89 ± 2.29	6.87 ± 2.30	7.03 ± 2.23	0.496
C-reactive protein (CRP), mg/L	7.07 ± 19.37	5.09 ± 13.23	18.50 ± 37.27	< 0.001
Albumin (Alb), g/L	39.41 ± 5.08	39.48 ± 4.79	38.98 ± 6.48	0.440
High-density lipoprotein cholesterol (HDL-C), mmol/L	1.14 ± 0.27	1.14 ± 0.27	1.16 ± 0.25	0.311
Fasting blood glucose (FBG), mmol/L	6.34 ± 2.60	6.34 ± 2.59	6.38 ± 2.70	0.864
Lipoprotein(a) (Lp(a)), mg/dL	24.34 ± 19.98	22.27 ± 17.75	36.32 ± 26.89	< 0.001
Systemic inflammation response index (SIRI), index	1.68 ± 3.20	1.66 ± 3.42	1.77 ± 1.43	0.558
Neutrophil-to-lymphocyte ratio (NLR), index	3.69 ± 6.06	3.65 ± 6.45	3.89 ± 2.87	0.518
Monocyte-to-lymphocyte ratio (MLR), index	0.32 ± 0.27	0.31 ± 0.29	0.33 ± 0.19	0.305
Derived neutrophil-to-lymphocyte ratio (dNLR), index	3.14 ± 8.53	2.75 ± 6.75	5.39 ± 15.04	0.075
C-reactive protein-to-albumin ratio (CAR), index	0.19 ± 0.53	0.13 ± 0.35	0.50 ± 1.05	< 0.001
Monocyte-to-high-density lipoprotein ratio (MHR), index	0.43 ± 0.38	0.43 ± 0.40	0.40 ± 0.18	0.282
Glucose-to-lymphocyte ratio (GLR), index	4.60 ± 3.00	4.54 ± 2.97	4.95 ± 3.20	0.216

Continuous variables are presented as mean ± SD and categorical variables as *n* (%). *P* values were calculated using Student’s t test for continuous variables and the χ² test (or Fisher’s exact test, as appropriate) for categorical variables

### Associations of Lp(a) and inflammatory markers with adverse outcomes

In multivariable logistic regression analyses (Table 2), higher Lp(a) levels were consistently associated with an increased risk of poor functional outcomes at both 14 and 90 days. After full adjustment, each 1-SD increase in Lp(a) was associated with a 42% higher odds of poor outcome at 14 days (OR 1.42, 95% CI 1.17–1.72) and a more than twofold higher odds at 90 days (OR 2.12, 95% CI 1.70–2.66). Patients in the highest tertile of Lp(a) had markedly increased risks compared with the lowest tertile (14-day: OR 2.39, 95% CI 1.52–3.81; 90-day: OR 2.74, 95% CI 1.52–5.07).

**Table 2:** Relationship between Different inflammatory markers and 14-day and 90-day poor prognosis

Exposure	Contrast	Crude OR (95% CI)	*P*	Model 1 OR (95% CI)	*P*	Model 2 OR (95% CI)	*P*
14-day poor prognosis							
Lpa	Per 1-SD increase (Z-score)	1.55 (1.33–1.82)	< 0.001	1.57 (1.33–1.86)	< 0.001	1.42 (1.17–1.72)	< 0.001
	Tertile 2 vs. 1	1.16 (0.77–1.74)	0.470	1.18 (0.77–1.83)	0.443	1.29 (0.81–2.07)	0.285
	Tertile 3 vs. 1	2.60 (1.78–3.84)	< 0.001	2.57 (1.70–3.90)	< 0.001	2.39 (1.52–3.81)	< 0.001
SIRI	Per 1-SD increase (Z-score)	1.06 (0.91–1.25)	0.428	1.03 (0.86–1.20)	0.746	0.92 (0.74–1.09)	0.381
	Tertile 2 vs. 1	0.96 (0.65–1.42)	0.841	1.12 (0.73–1.71)	0.606	1.06 (0.67–1.67)	0.806
	Tertile 3 vs. 1	1.76 (1.21–2.57)	0.003	1.63 (1.08–2.46)	0.020	1.12 (0.71–1.77)	0.633
NLR	Per 1-SD increase (Z-score)	1.03 (0.88–1.21)	0.649	0.99 (0.80–1.15)	0.861	0.87 (0.63–1.05)	0.251
	Tertile 2 vs. 1	1.09 (0.73–1.62)	0.685	0.99 (0.65–1.52)	0.974	0.98 (0.62–1.55)	0.926
	Tertile 3 vs. 1	2.08 (1.43–3.05)	< 0.001	1.68 (1.11–2.55)	0.014	1.09 (0.68–1.74)	0.714
MLR	Per 1-SD increase (Z-score)	1.07 (0.92–1.26)	0.354	1.04 (0.87–1.22)	0.612	1.00 (0.80–1.19)	0.986
	Tertile 2 vs. 1	0.79 (0.53–1.16)	0.230	0.74 (0.48–1.12)	0.154	0.65 (0.41–1.03)	0.068
	Tertile 3 vs. 1	1.51 (1.04–2.19)	0.030	1.23 (0.82–1.86)	0.309	1.18 (0.75–1.85)	0.472
dNLR	Per 1-SD increase (Z-score)	1.51 (1.12–2.28)	0.035	1.51 (1.14–2.26)	0.023	1.26 (1.03–1.70)	0.067
	Tertile 2 vs. 1	0.86 (0.57–1.29)	0.470	0.90 (0.58–1.38)	0.620	0.96 (0.60–1.52)	0.853
	Tertile 3 vs. 1	2.24 (1.54–3.28)	< 0.001	2.03 (1.36–3.05)	< 0.001	1.39 (0.89–2.18)	0.151
CAR	Per 1-SD increase (Z-score)	1.93 (1.46–2.63)	< 0.001	2.00 (1.48–2.78)	< 0.001	1.56 (1.21–2.20)	0.004
	Tertile 2 vs. 1	1.02 (0.69–1.52)	0.920	0.91 (0.60–1.39)	0.668	0.87 (0.54–1.39)	0.560
	Tertile 3 vs. 1	1.86 (1.28–2.72)	0.001	1.71 (1.14–2.58)	0.010	1.68 (1.07–2.66)	0.024
MHR	Per 1-SD increase (Z-score)	0.85 (0.64–1.03)	0.179	0.94 (0.71–1.14)	0.631	0.92 (0.66–1.15)	0.567
	Tertile 2 vs. 1	0.79 (0.54–1.15)	0.212	0.96 (0.64–1.44)	0.835	0.91 (0.58–1.42)	0.677
	Tertile 3 vs. 1	0.80 (0.55–1.17)	0.251	1.03 (0.68–1.56)	0.892	0.95 (0.60–1.51)	0.836
GLR	Per 1-SD increase (Z-score)	1.18 (1.02–1.38)	0.029	1.13 (0.96–1.33)	0.126	1.03 (0.86–1.24)	0.718
	Tertile 2 vs. 1	1.06 (0.72–1.57)	0.766	0.99 (0.65–1.51)	0.975	0.99 (0.63–1.58)	0.982
	Tertile 3 vs. 1	1.53 (1.05–2.23)	0.028	1.29 (0.86–1.94)	0.219	1.07 (0.68–1.70)	0.765
90-day poor prognosis							
Lpa	Per 1-SD increase (Z-score)	2.20 (1.83–2.67)	< 0.001	2.33 (1.91–2.86)	< 0.001	2.12 (1.70–2.66)	< 0.001
	Tertile 2 vs. 1	1.15 (0.63–2.12)	0.645	1.15 (0.62–2.15)	0.660	1.30 (0.67–2.54)	0.435
	Tertile 3 vs. 1	3.36 (2.02–5.79)	< 0.001	3.19 (1.87–5.59)	< 0.001	2.74 (1.52–5.07)	0.001
SIRI	Per 1-SD increase (Z-score)	1.03 (0.81–1.21)	0.735	1.01 (0.76–1.19)	0.954	0.83 (0.51–1.09)	0.337
	Tertile 2 vs. 1	0.96 (0.56–1.66)	0.890	1.15 (0.65–2.03)	0.632	1.05 (0.57–1.94)	0.869
	Tertile 3 vs. 1	1.79 (1.10–2.96)	0.021	1.72 (1.02–2.92)	0.043	0.96 (0.53–1.76)	0.899
NLR	Per 1-SD increase (Z-score)	1.04 (0.81–1.21)	0.701	1.00 (0.74–1.19)	0.975	0.75 (0.42–1.07)	0.262
	Tertile 2 vs. 1	0.76 (0.43–1.32)	0.325	0.70 (0.39–1.24)	0.222	0.64 (0.34–1.18)	0.156
	Tertile 3 vs. 1	1.75 (1.09–2.86)	0.023	1.43 (0.86–2.41)	0.173	0.75 (0.41–1.38)	0.359
MLR	Per 1-SD increase (Z-score)	1.07 (0.88–1.26)	0.438	1.06 (0.82–1.27)	0.596	0.96 (0.69–1.23)	0.794
	Tertile 2 vs. 1	1.19 (0.71-2.00)	0.511	1.19 (0.70–2.03)	0.530	1.09 (0.60–1.96)	0.782
	Tertile 3 vs. 1	1.39 (0.84–2.31)	0.203	1.15 (0.67–1.97)	0.620	0.90 (0.50–1.63)	0.725
dNLR	Per 1-SD increase (Z-score)	1.22 (1.04–1.53)	0.030	1.24 (1.04–1.58)	0.031	1.16 (0.97–1.40)	0.083
	Tertile 2 vs. 1	0.53 (0.29–0.94)	0.034	0.56 (0.30-1.00)	0.055	0.59 (0.31–1.11)	0.106
	Tertile 3 vs. 1	1.66 (1.04–2.67)	0.035	1.45 (0.89–2.39)	0.136	0.86 (0.49–1.51)	0.595
CAR	Per 1-SD increase (Z-score)	1.95 (1.49–2.61)	< 0.001	2.02 (1.52–2.77)	< 0.001	1.59 (1.28–2.13)	< 0.001
	Tertile 2 vs. 1	0.96 (0.55–1.69)	0.886	0.90 (0.50–1.61)	0.715	0.89 (0.47–1.71)	0.737
	Tertile 3 vs. 1	2.14 (1.31–3.56)	0.003	2.03 (1.21–3.45)	0.008	2.22 (1.25–4.06)	0.008
MHR	Per 1-SD increase (Z-score)	0.91 (0.63–1.13)	0.517	1.02 (0.73–1.25)	0.878	1.03 (0.68–1.30)	0.867
	Tertile 2 vs. 1	0.87 (0.53–1.45)	0.606	1.13 (0.66–1.94)	0.657	1.06 (0.59–1.91)	0.854
	Tertile 3 vs. 1	1.03 (0.63–1.69)	0.900	1.39 (0.82–2.37)	0.216	1.29 (0.71–2.35)	0.404
GLR	Per 1-SD increase (Z-score)	1.13 (0.93–1.35)	0.193	1.08 (0.88–1.31)	0.422	0.94 (0.72–1.20)	0.659
	Tertile 2 vs. 1	0.77 (0.45–1.32)	0.345	0.70 (0.40–1.21)	0.204	0.65 (0.35–1.21)	0.179
	Tertile 3 vs. 1	1.35 (0.84–2.20)	0.223	1.11 (0.67–1.85)	0.681	0.92 (0.52–1.63)	0.772

Crude model: unadjusted. Model 1: adjusted for age, sex, body mass index (BMI), smoking status, alcohol consumption, and physical activity. Model 2: further adjusted for hypertension, diabetes, heart disease, use of antihypertensive and antidiabetic drugs, TOAST classification, and admission NIHSS score. OR, odds ratio; CI, confidence interval. Z-score indicates per 1-SD increase. Tertile comparisons use tertile 1 as the reference group. *P*-values < 0.001 are shown as < 0.001

Among inflammatory indices, CAR showed the strongest and most consistent association with outcomes. In the fully adjusted model, each 1-SD increase in CAR was associated with higher odds of poor prognosis at 14 days (OR 1.56, 95% CI 1.21–2.20) and 90 days (OR 1.59, 95% CI 1.28–2.13). Participants in the highest CAR tertile also had significantly increased risks compared with the lowest tertile (14-day: OR 1.68, 95% CI 1.07–2.66; 90-day: OR 2.22, 95% CI 1.25–4.06). In contrast, other inflammatory indices (SIRI, NLR, MLR, MHR, and GLR) were not significantly associated with outcomes after multivariable adjustment.

### Predictive performance of Lp(a) and inflammatory markers

**Fig. 2:**
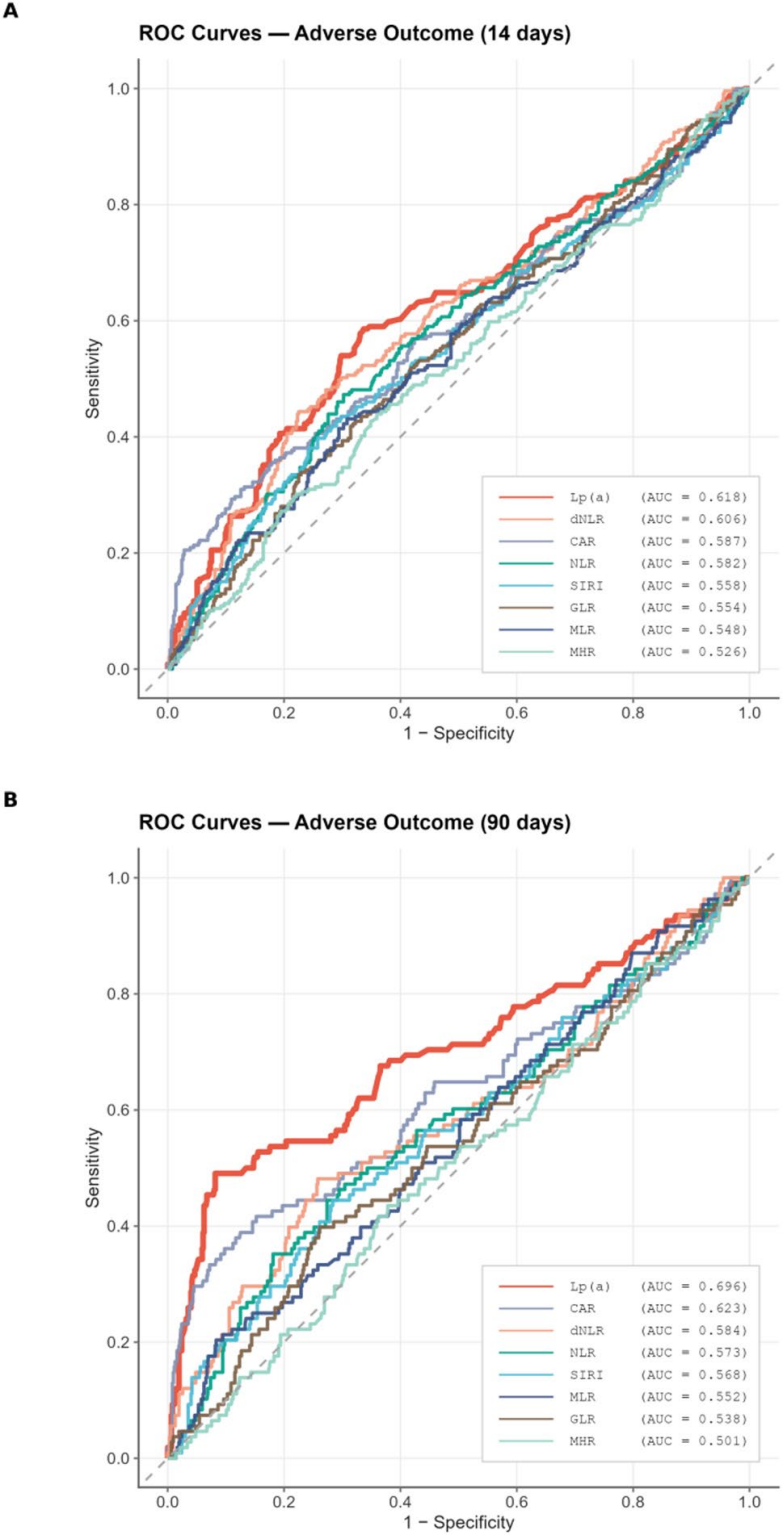
Receiver operating characteristic (ROC) curves of inflammatory markers for predicting 14-day and 90-day poor prognosis..Receiver operating characteristic (ROC) curve analysis comparing the discriminative performance of multiple predictive indicators for poor prognosis at 14 days (Panel **A**) and 90 days (Panel **B**). The x-axis represents 1 − specificity, and the y-axis represents sensitivity. The dashed diagonal line indicates no discriminative ability. The colored curves represent the individual predictive indicators, including Lp(a), NLR, dNLR, MHR, SIRI, MLR, CAR, and GLR, with AUC values shown in the legends

Receiver operating characteristic analyses showed that Lp(a) had the highest AUC among the evaluated biomarkers for adverse outcomes, although its absolute discriminatory performance remained modest. For the 14-day outcome, the area under the curve (AUC) for Lp(a) was 0.618, exceeding that of CAR (0.587), NLR (0.582), SIRI (0.558), GLR (0.554), MLR (0.548), dNLR (0.606), and MHR (0.526) (Fig. 2). A similar pattern was observed for the 90-day outcome, with Lp(a) achieving the highest AUC (0.696), followed by CAR (0.623), whereas the remaining inflammatory indices showed lower discriminatory performance (AUC range 0.501–0.584).

**Fig. 3:**
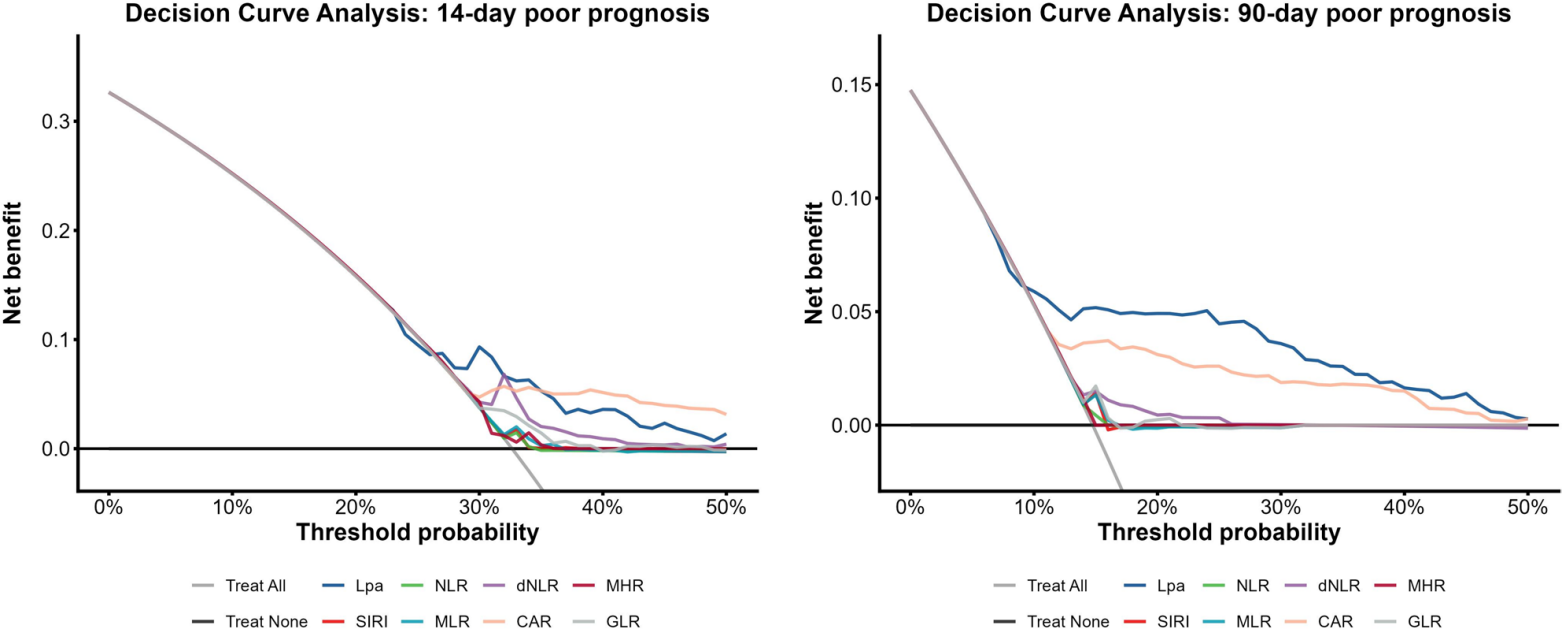
Decision Curve Analysis of Predictive Models for 14-day and 90-day Poor Prognosis. Decision curve analysis (DCA) comparing the clinical net benefit of multiple predictive models for poor prognosis at 14 days (left panel) and 90 days (right panel). The x-axis represents the threshold probability, and the y-axis represents the net benefit. The gray line indicates the strategy of treating all patients (Treat All), and the black horizontal line indicates treating none (Treat None). The colored curves represent the individual predictive models (Lpa, NLR, dNLR, MHR, SIRI, MLR, CAR, and GLR). A model is considered clinically useful when it yields a higher net benefit than both the “treat all” and “treat none” strategies across a range of threshold probabilities

Decision curve analysis further suggested that Lp(a) produced the highest clinical net benefit over diverse decision threshold levels for the prediction of both 14-day and 90-day outcomes. CAR also showed modest clinical utility, whereas other inflammatory markers yielded minimal incremental net benefit compared with the treat-all or treat-none strategies (Fig. 3).

### Comparative predictive performance of Lp(a) versus individual inflammatory markers

Lp(a) demonstrated consistently better risk discrimination than individual inflammatory markers for both short- and medium-term outcomes (Table 3). For the 14-day outcome, the AUC of Lp(a) exceeded that of each inflammatory marker when evaluated in pairwise comparisons, with absolute differences ranging from 0.012 to 0.092. Correspondingly, discrimination and reclassification metrics also favored Lp(a), with IDI values ranging from 0.030 to 0.045 and NRI values ranging from 0.197 to 0.385 across the comparisons (all *P* < 0.001 except for the IDI comparison with CAR).

**Table 3:** Comparative predictive performance of Lp(a) versus individual inflammatory markers

Outcome	Reference (Inflammation)	AUC (Inflammation)	AUC [Lp(a)]	ΔAUC	IDI (95% CI)	*P*	NRI (95% CI)	*P*
14-day poor prognosis	SIRI	0.558	0.618	0.060	0.045 (0.028, 0.061)	**< 0.001**	0.364 (0.217, 0.508)	**< 0.001**
14-day poor prognosis	NLR	0.582	0.618	0.036	0.045 (0.028, 0.061)	**< 0.001**	0.385 (0.228, 0.539)	**< 0.001**
14-day poor prognosis	MLR	0.548	0.618	0.070	0.044 (0.026, 0.061)	**< 0.001**	0.372 (0.222, 0.515)	**< 0.001**
14-day poor prognosis	dNLR	0.606	0.618	0.012	0.030 (0.013, 0.047)	**< 0.001**	0.352 (0.196, 0.504)	**< 0.001**
14-day poor prognosis	CAR	0.587	0.618	0.031	-0.005 (-0.024, 0.014)	0.590	0.197 (0.043, 0.344)	**0.014**
14-day poor prognosis	MHR	0.526	0.618	0.092	0.043 (0.025, 0.061)	**< 0.001**	0.352 (0.207, 0.507)	**< 0.001**
14-day poor prognosis	GLR	0.554	0.618	0.064	0.039 (0.023, 0.058)	**< 0.001**	0.378 (0.223, 0.528)	**< 0.001**
90-day poor prognosis	SIRI	0.568	0.696	0.128	0.135 (0.097, 0.174)	**< 0.001**	0.702 (0.500, 0.906)	**< 0.001**
90-day poor prognosis	NLR	0.573	0.696	0.123	0.135 (0.099, 0.174)	**< 0.001**	0.699 (0.504, 0.909)	**< 0.001**
90-day poor prognosis	MLR	0.552	0.696	0.145	0.134 (0.096, 0.176)	**< 0.001**	0.709 (0.508, 0.911)	**< 0.001**
90-day poor prognosis	dNLR	0.584	0.696	0.112	0.125 (0.089, 0.168)	**< 0.001**	0.665 (0.466, 0.878)	**< 0.001**
90-day poor prognosis	CAR	0.623	0.696	0.073	0.065 (0.029, 0.100)	**< 0.001**	0.507 (0.311, 0.699)	**< 0.001**
90-day poor prognosis	MHR	0.501	0.696	0.195	0.134 (0.099, 0.174)	**< 0.001**	0.686 (0.489, 0.884)	**< 0.001**
90-day poor prognosis	GLR	0.538	0.696	0.158	0.133 (0.095, 0.172)	**< 0.001**	0.683 (0.483, 0.887)	**< 0.001**

Table Pairwise comparison of Lp(a) versus each inflammatory indicator. Reference model: single inflammatory marker (Z-scored), New model: Lp(a) (Z-scored). *IDI*  Integrated Discrimination Improvement, *NRI*  Net Reclassification Improvement (continuous). 95% CI and *P* values derived from 1000 bootstrap iterations. Bold *P* values indicate statistical significance (two-sided *P* < 0.05). Blue rows indicate Lp(a) achieves higher AUC (ΔAUC > 0). Abbreviations: *IDI* Integrated Discrimination Improvement, *NRI* Net Reclassification Improvement, Δ*AUC*  AUC[Lp(a)] − AUC[Inflammation marker]

The superiority of Lp(a) was more pronounced for the 90-day outcome. In pairwise comparisons with individual inflammatory markers, the AUC advantage of Lp(a) ranged from 0.073 to 0.195. Consistent improvements were also observed in IDI (0.065–0.135) and NRI (0.507–0.709) across the corresponding comparisons (all *P* < 0.001). These findings indicate that Lp(a) provides stronger prognostic discrimination than commonly used inflammatory indices.

### WQS analysis of Lp(a), inflammatory markers, and poor 90-day outcome

**Fig. 4:**
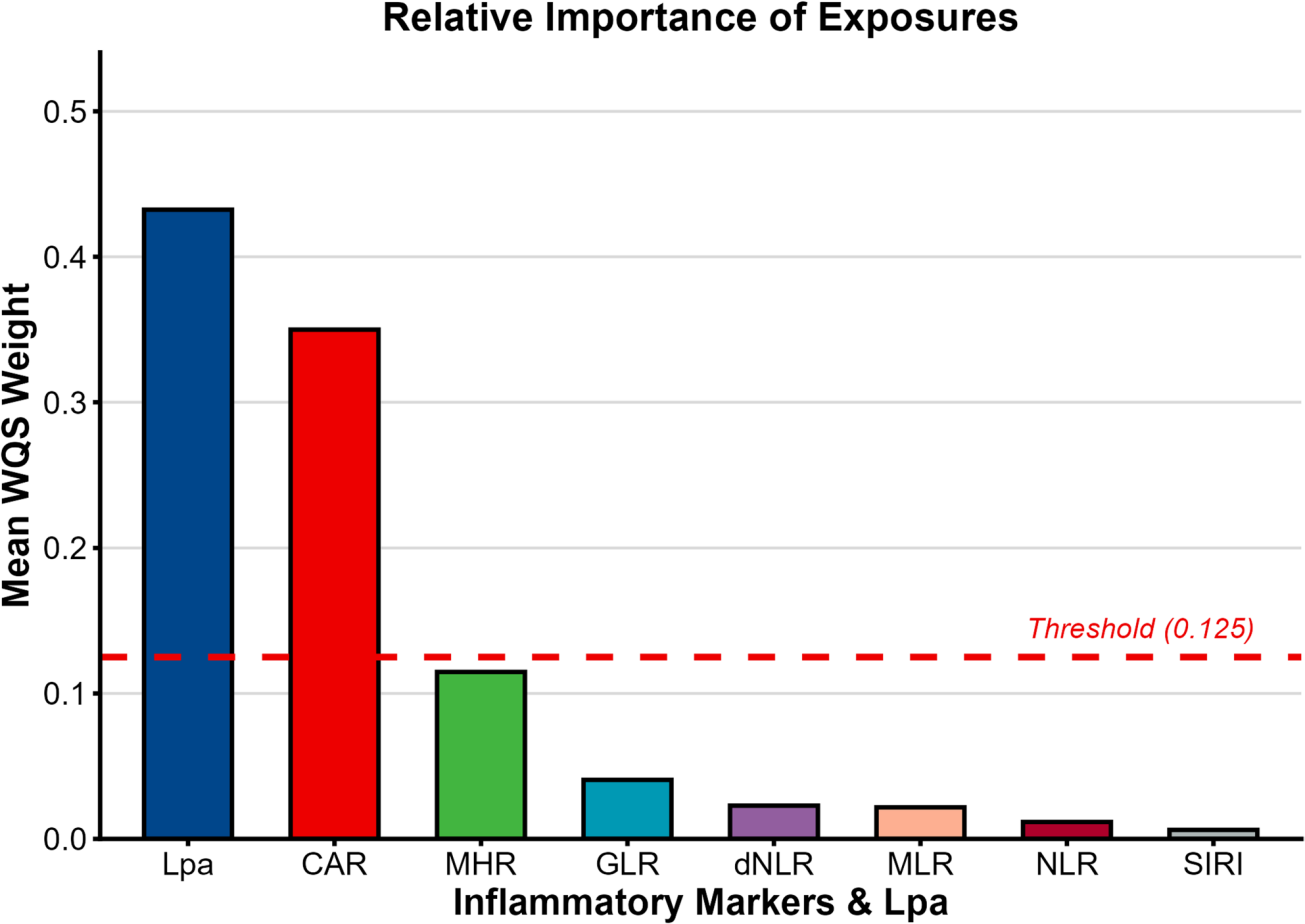
WQS Weight Distribution for Inflammatory Markers & Lp(a). The weights represent the contribution of each variable to the model, with higher values indicating more significant contributions. The markers are plotted according to their weight, providing an overview of the relative importance of each factor in the model

In the forward WQS analysis (Fig. 4), the overall positive association of the biomarker mixture with poor outcome was driven predominantly by Lp(a) and CAR. Lp(a) showed the largest contribution, with an average weight of 0.43, followed by CAR with a weight of 0.35; both clearly exceeded the prespecified threshold of 0.125, indicating that they were the principal components underlying the mixture effect. By comparison, MHR made only a modest contribution, with a weight of 0.11, which approached but did not clearly surpass the threshold. The remaining inflammatory indices, including GLR, dNLR, MLR, NLR, and SIRI, contributed minimally to the mixture, with all weights below 0.05. Taken together, these findings suggest that the positive joint association observed for the inflammation-related marker-Lp(a) mixture was largely attributable to Lp(a) and CAR, whereas the other inflammatory markers provided little additional contribution to the overall signal.

### Joint association of CAR and Lp(a) with poor prognosis

Using the low CAR/low Lp(a) group as the reference, patients with concurrent elevation of both markers exhibited the highest risk of poor outcome at both 14 days (adjusted OR, 3.36; 95% CI, 1.98–5.77) and 90 days (adjusted OR, 4.60; 95% CI, 2.29–9.81). At 14 days, elevated Lp(a) alone was also associated with an increased risk of poor prognosis (adjusted OR, 2.26; 95% CI, 1.31–3.94), whereas isolated elevation of CAR showed only a modest and non-significant association (adjusted OR, 1.45; 95% CI, 0.83–2.53). By contrast, the combination of high CAR and high Lp(a) produced the largest effect estimates, indicating a clear gradient of increasing risk across exposure categories.

**Fig. 5:**
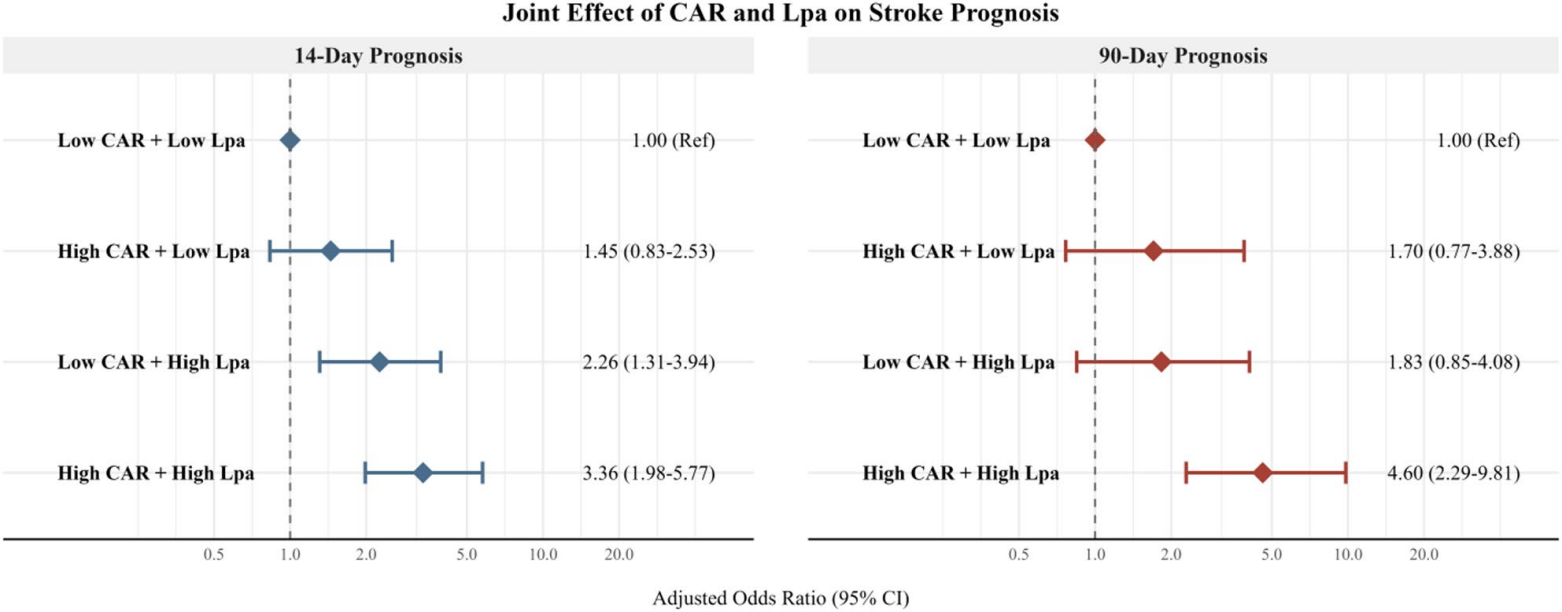
Joint association of CAR and Lp(a) with 14-day and 90-day poor prognosis. Using the low CAR + low Lp(a) group as the reference, the adjusted odds ratios (95% confidence intervals) for adverse outcomes at 14 days and 90 days in the combined CAR and Lp(a) classification are shown. Adjusted for covariates: age, sex, body mass index (BMI), smoking status, alcohol consumption, physical activity, hypertension, diabetes, heart disease, use of antihypertensive and antidiabetic drugs, TOAST classification, and admission NIHSS score

At 90 days, the excess risk remained most pronounced among patients with concurrent elevation of both biomarkers, while the groups with isolated elevation of either marker showed smaller and statistically non-significant associations. These patterns suggest that the adverse prognostic impact of CAR and Lp(a) may become more apparent when both markers are elevated simultaneously, highlighting a potential joint effect of inflammatory burden and atherogenic lipid pathways on post-stroke recovery (Fig. 5).

### Mediation analysis of CAR in the association between Lp(a) and 90-day adverse outcome

In an exploratory mediation analysis allowing for exposure–mediator interaction, the association between Lp(a) and 90-day adverse outcome was statistically decomposed into a larger direct-effect estimate and a smaller indirect-effect estimate via CAR (Supplementary Fig. 1). The total effect was significant (estimate, 0.050; 95% CI, 0.033–0.074; *P* < 0.001), as were the direct effects at both treated and control levels of Lp(a) (ADE, 0.046; 95% CI, 0.030–0.066; and 0.041; 95% CI, 0.026–0.059, respectively; both *P* < 0.001). The indirect effect through CAR was significant at the treated level (ACME, 0.008; 95% CI, 0.002–0.026; *P* = 0.011), but not at the control level (ACME, 0.004; 95% CI, − 0.001 to 0.019; *P* = 0.089). Overall, these findings suggest that CAR may statistically account for a limited proportion of the association between Lp(a) and 90-day outcome; however, given the observational design and concurrent baseline measurement of Lp(a) and CAR, they should not be interpreted as definitive evidence of a causal mediating mechanism.

### Subgroup analyses of the association between Lp(a) and 90-day poor functional outcome

Per 1-SD increase in Lp(a), the risk of 90-day poor functional outcome was increased across most prespecified subgroups, including all age tertiles and both sexes (Supplementary Fig. 2). The association was strongest in the youngest age tertile (OR, 2.31; 95% CI, 1.31–4.42) and in female (OR, 1.89; 95% CI, 1.34–2.71), but there was no significant interaction by age, sex, or smoking status (all P for interaction > 0.05). This indicates that the overall effect of Lp(a) is broadly consistent across all subgroups.

### Sensitivity analysis

After FDR correction(Table S1), the associations of Lp(a) with both 14-day and 90-day poor prognosis remained significant in both the per-SD and tertile analyses, whereas CAR remained significant for 14-day prognosis only in the per-SD model and for 90-day prognosis in both models. No other inflammatory marker survived multiple-testing correction. E-values for Lp(a) and CAR were modest but consistently above 1, supporting the relative robustness of the observed associations to unmeasured confounding.

IPTW analyses yielded results broadly consistent with the primary models(Table S2). In the fully adjusted IPTW model, CAR remained associated with both 14-day poor prognosis (OR, 2.19; 95% CI, 1.33–3.59) and 90-day poor prognosis (OR, 2.04; 95% CI, 1.29–3.24), particularly in the per-SD analysis. Lp(a) remained associated with 90-day poor prognosis in both the per-SD analysis (OR, 1.53; 95% CI, 1.24–1.88) and the highest-versus-lowest tertile comparison (OR, 2.37; 95% CI, 1.37–4.08), and with 14-day poor prognosis in the highest-versus-lowest tertile comparison (OR, 1.73; 95% CI, 1.16–2.58). No other inflammatory marker showed a robust association after full IPTW adjustment.

Finally, in an additional sensitivity analysis that re-included the 22 patients who underwent reperfusion therapy for the index event, the main findings remained materially unchanged after further adjustment for reperfusion therapy status (Table S3). In the fully adjusted model, each 1-SD increase in Lp(a) remained associated with both 14-day poor prognosis (OR, 1.46; 95% CI, 1.20–1.78) and 90-day poor prognosis (OR, 2.23; 95% CI, 1.77–2.82). Similarly, each 1-SD increase in CAR remained associated with both 14-day poor prognosis (OR, 1.61; 95% CI, 1.24–2.27) and 90-day poor prognosis (OR, 1.66; 95% CI, 1.31–2.25). The corresponding tertile analyses showed a similar pattern, indicating that re-inclusion of reperfusion-treated patients did not materially alter the principal conclusions.

## Discussion

In this prospective cohort study of elderly patients with AIS, elevated Lp(a) levels were significantly associated with poorer functional outcomes at both 14 days and 90 days, with a stronger association at the 90-day endpoint. Among the inflammatory markers assessed, only CAR maintained a consistent and independent association with poor outcomes after multivariate adjustment, while other markers contributed minimally after accounting for clinical covariates. In comparative analyses, Lp(a) had the highest AUC among the biomarkers evaluated, particularly for the 90-day outcome; however, its absolute discriminatory performance remained modest. WQS analysis indicated that Lp(a) and CAR jointly explained the majority of prognostic signals, with patients exhibiting elevated levels of both markers carrying the highest risk of poor outcomes. Exploratory mediation analysis suggested that the association between Lp(a) and 90-day adverse outcome could be statistically decomposed into a larger direct-effect estimate and a smaller indirect-effect estimate via CAR. Collectively, these findings suggest that Lp(a) may be a relatively more informative biomarker than the individual inflammatory indices evaluated in this study, while CAR appears to be the inflammatory marker most closely associated with adverse prognosis.

Our findings are consistent with some previous studies reporting an adverse prognostic association of elevated Lp(a) after ischemic stroke. A prospective study of 973 patients with acute ischemic stroke reported that higher Lp(a) was associated with major disability or death at 6 months, including when Lp(a) was discordantly elevated despite lower LDL-C, supporting the concept that Lp(a) captures residual vascular risk beyond conventional lipid measures [28]. In parallel, a recent meta-analysis found that CAR was consistently associated with poor functional outcome and mortality in stroke, reinforcing the value of inflammation-related composite markers in prognostic assessment [18]. However, the literature is not entirely uniform. Earlier studies have reported null findings regarding the prognostic role of Lp(a). For example, van Kooten et al. found no clear relationship between admission Lp(a) levels and stroke severity or prognosis in acute cerebral ischemia [29], and More et al. likewise reported no significant correlation between Lp(a) levels and stroke severity [30], in-hospital outcome, or mortality. These discrepancies may reflect differences in study design, sample size, patient characteristics, follow-up duration, outcome definitions, assay methods, and the timing of biomarker measurement.

Several biological mechanisms may explain why Lp(a) and CAR are linked to post-stroke functional recovery. Lp(a) is structurally similar to LDL but contains apolipoprotein(a), which allows it to carry oxidized phospholipids and interact with fibrinolytic pathways [31, 32]. These properties may promote endothelial dysfunction, vascular inflammation, and prothrombotic activity, thereby exacerbating microvascular injury and impairing reperfusion in the ischemic brain [33]. In addition, Lp(a) has been implicated in amplifying thrombo-inflammatory signaling, which may contribute to infarct expansion and delayed neurological recovery after the acute event [34, 35]. In this context, CAR may be particularly informative in the early phase after stroke because it integrates two complementary biological dimensions: acute systemic inflammatory activation, reflected by CRP, and nutritional/metabolic reserve, reflected by albumin [18]. Elevated CRP indicates activation of the acute-phase response and systemic inflammation, whereas lower albumin levels may reflect catabolic stress, oxidative burden, and impaired vascular protection [36, 37]. Together, these processes may aggravate blood–brain barrier disruption, intensify neuroinflammation, and limit the capacity for post-stroke repair, thereby contributing to poorer functional outcomes [38, 39].

Our results align with these observations, showing independent associations of both Lp(a) and CAR with adverse functional recovery; however, unlike much of the previous literature that evaluated either Lp(a) or CAR in isolation, we assessed them together against a broader panel of inflammatory indices and found that the prognostic signal was concentrated largely in Lp(a) and CAR, whereas markers such as SIRI, NLR, MLR, MHR, and GLR were attenuated after multivariable adjustment. This pattern suggests that, in older patients with AIS, composite indices integrating acute-phase response and systemic metabolic stress may better reflect clinically relevant inflammatory burden than leukocyte-derived ratios alone.

Our findings regarding the joint effect of Lp(a) and inflammation are also directionally consistent with cardiovascular studies showing that higher inflammatory burden can amplify Lp(a)-related risk in high-risk settings, including cohorts undergoing percutaneous coronary intervention and patients with acute myocardial infarction [13]. From a biological perspective, this interaction is plausible because Lp(a) participates in inflammatory signaling pathways while inflammation itself may enhance the pathogenicity of oxidized phospholipids carried by Lp(a), creating a feed-forward loop that aggravates vascular injury [40]. At the same time, our exploratory mediation analysis suggested that the association between Lp(a) and poor 90-day outcome was characterized by a larger direct-effect estimate and a smaller indirect-effect estimate via CAR. However, because unmeasured mediator–outcome confounding cannot be excluded and because Lp(a) and CAR were measured concurrently at baseline, these findings should be interpreted cautiously as hypothesis-generating rather than as definitive evidence of a causal mechanism.

Several factors may explain these apparent differences across studies. First, study populations differed substantially: prior interaction studies were conducted mainly in coronary disease or acute myocardial infarction, whereas our cohort comprised older patients with AIS, in whom neuroinflammation, infarct evolution, frailty, and nutritional reserve may modify biomarker-performance relationships [7, 12, 13, 41]. Second, outcome definitions and follow-up windows were not the same; studies focused on cardiovascular mortality or recurrent coronary events are not directly comparable with short-term functional recovery after stroke [42]. Third, inflammatory phenotypes were characterized differently: many prior cardiovascular studies relied on hsCRP alone, whereas CAR integrates CRP and albumin and may therefore capture a broader inflammatory and systemic stress profile than hsCRP alone [36, 37]. Finally, differences in age structure, ethnicity, baseline stroke severity, assay methods, covariate adjustment, and the timing of biomarker measurement may all have contributed to heterogeneity across studies [42, 43].

In this context, our data suggest that the inflammation-Lp(a) axis is relevant in AIS, but that its clinical expression may be more additive than strongly interaction-dependent [42], with CAR emerging as the inflammatory indicator most closely aligned with Lp(a)-related prognostic risk in this setting. Our manuscript’s own results support this interpretation: Lp(a) remained consistently associated with poor outcome at both 14 and 90 days, CAR was the only inflammatory index to show similarly robust adjusted associations, and the mixture and joint analyses indicated that most of the combined prognostic signal was carried by Lp(a) and CAR rather than by the other inflammatory indices.

The strengths of this study include its prospective design and the simultaneous evaluation of a comprehensive panel of inflammation-related indices alongside Lp(a), which enabled a direct comparison of their relative prognostic utility. An additional strength is that all participants were enrolled within 72 h of symptom onset and baseline biomarkers were collected within 24 h of admission under standardized fasting conditions, which likely reduced, although did not eliminate, confounding from later post-stroke complications such as infection, prolonged stress responses, and nutritional deterioration. Furthermore, the application of an advanced analytical framework—including weighted quantile sum regression to assess mixture effects and counterfactual mediation analysis allowing for exposure-mediator interactions—provided nuanced insights into the interplay between lipid and inflammatory profiles. The robustness of these findings is additionally supported by consistent results across continuous and categorical regression analyses, inverse probability of treatment weighting (IPTW), and multiple-testing sensitivity analyses. Nevertheless, several limitations should be acknowledged. First, the single-center design and the restriction to older Chinese patients with AIS may limit the generalizability of our results to younger populations or different ethnic groups. In addition, because patients receiving reperfusion therapy were excluded from the primary analytic cohort to reduce treatment-related heterogeneity, the primary findings may not be fully generalizable to AIS patients treated with modern acute-stage reperfusion interventions. However, sensitivity analyses re-including these patients yielded materially similar estimates, supporting the robustness of the main associations. Second, biomarker concentrations were assessed only at admission; therefore, the dynamic trajectories of Lp(a) and CAR during the acute and subacute phases of stroke, as well as their relationships with evolving neurological outcomes, remain uncharacterized. This limitation is particularly relevant to interpretation of the mediation analysis, because Lp(a) and CAR were measured concurrently at baseline rather than sequentially, and the presumed temporal ordering between Lp(a), CAR, and outcome could therefore not be directly established. In addition, counterfactual mediation analysis relies on the assumption that there is no unmeasured confounding of the mediator–outcome relationship, an assumption that may not be fully satisfied in our study because CAR can be influenced by factors not comprehensively captured in the dataset, such as occult infection severity, frailty, pre-stroke inflammatory status, nutritional reserve, and infarct-related clinical complexity. Accordingly, the mediation findings should be regarded as exploratory and hypothesis-generating rather than as definitive evidence of a causal mediating pathway. Third, despite extensive covariate adjustment and E-value estimates indicating relative resilience to unmeasured confounding, residual confounding from unmeasured factors—such as precise infarct volume, specific pre-stroke medication adherence, or underlying genetic variations—cannot be definitively excluded. Finally, our follow-up was limited to 90 days, precluding conclusions regarding long-term disability, cognitive decline, or recurrent cardiovascular events.

## Conclusion

In conclusion, elevated baseline Lp(a) was independently associated with poor early and 90-day functional outcomes in older patients with AIS, and CAR was the inflammatory index most consistently associated with adverse prognosis. Combined assessment of Lp(a) and CAR may be useful for early risk stratification, although further validation is required. Future multicenter studies with repeated biomarker measurements are needed to clarify the temporal interplay between Lp(a), inflammation, and post-stroke recovery. The mediation findings in the present study should be interpreted as exploratory and hypothesis-generating.

## Supplementary Information

Supplementary Material 1.

## Data Availability

Origin data of this study are available from the corresponding author on reasonable request.

## Abbreviations

AIS: acute ischemic stroke
Lp(a): Lipoprotein(a)
HDL: High-density lipoprotein
SIRI: Systemic inflammation response index
NLR: Neutrophil-to-lymphocyte ratio
MLR: Monocyte-to-lymphocyte ratio
dNLR: Derived neutrophil-to-lymphocyte ratio
CAR: C-reactive protein-to-albumin ratio
MHR: Monocyte-to-high-density lipoprotein ratio
GLR: Glucose-to-lymphocyte ratio
