# Supplementary material for: Association of lipoprotein(a) and composite inflammatory indices with functional outcomes in acute ischemic stroke: a prospective cohort study
Source: Lipids Health Dis. 2026 May 11;25:165. doi: 10.1186/s12944-026-02975-6 (PMC13330171; doi:10.1186/s12944-026-02975-6)
Supplement: Supplementary file 1 — Supplementary Material 1. [file 12944_2026_2975_MOESM1_ESM.docx]

**Supplementary Materials**

### **Calculation of inflammation-related composite indices**

All composite indices were calculated from baseline laboratory parameters. Neutrophil count (NC), lymphocyte count (LC), monocyte count (MONO), and white blood cell count (WBC) were obtained as absolute counts (×10^9/L). C-reactive protein (CRP) was recorded in mg/L, albumin (Alb) in g/L, high-density lipoprotein cholesterol (HDL-C) in mmol/L, and fasting blood glucose (FBG) in mmol/L.

The indices were computed as follows (using baseline values):

1.Systemic inflammation response index (SIRI) = (NC × MONO) / LC

2.Neutrophil-to-lymphocyte ratio (NLR) = NC / LC

3.Monocyte-to-lymphocyte ratio (MLR) = MONO / LC

4.Derived neutrophil-to-lymphocyte ratio (dNLR) = NC / (WBC − NC)

5.C-reactive protein-to-albumin ratio (CAR) = CRP / Alb

6.Monocyte-to-high-density lipoprotein ratio (MHR) = MONO / HDL-C

7.Glucose-to-lymphocyte ratio (GLR) = FBG / LC

All indices were calculated without additional unit conversion, using the laboratory-reported units specified above.


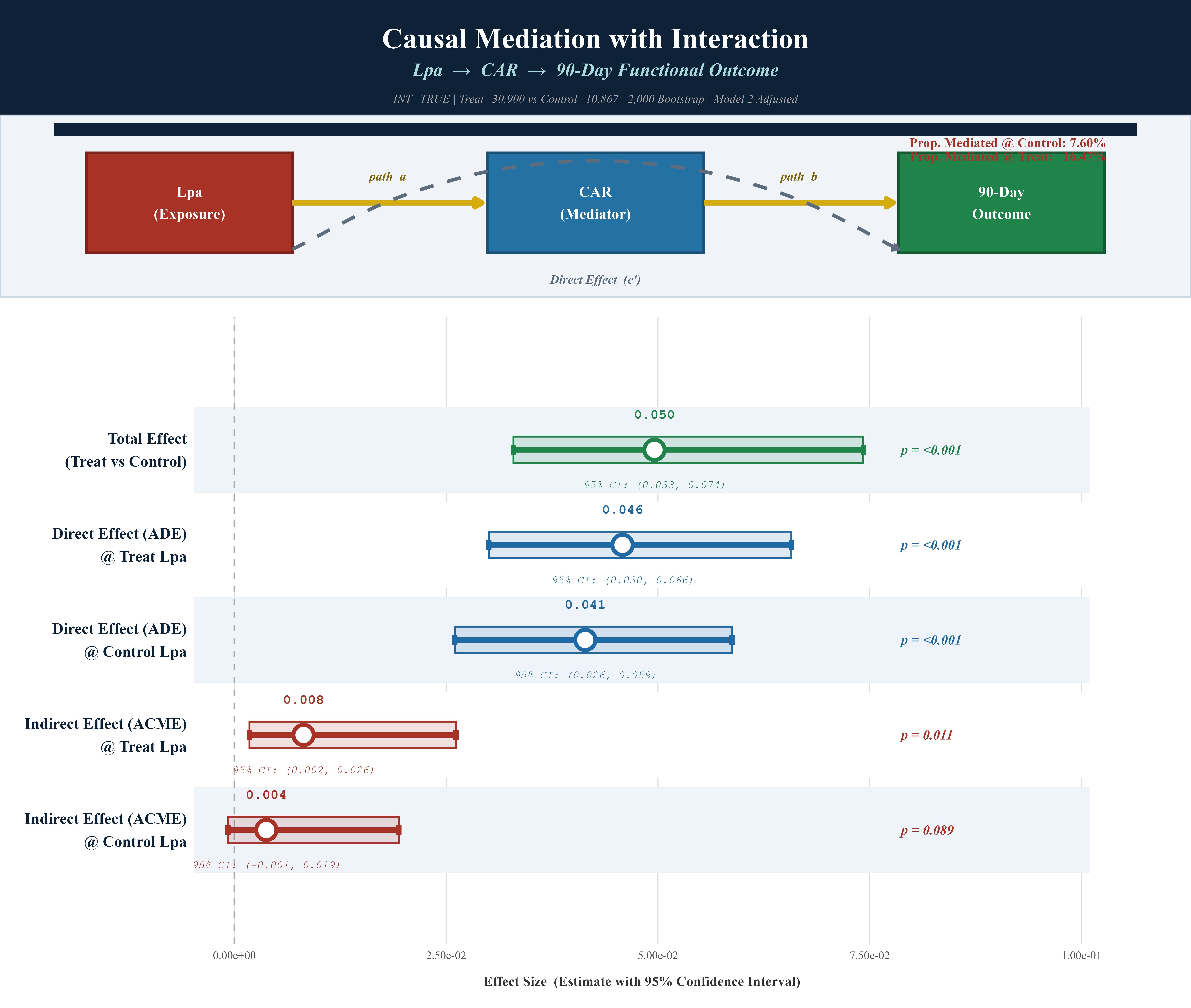


**Supplementary Fig. 1.** Exploratory mediation analysis of the association between Lp(a), CAR, and 90-day adverse outcome

Counterfactual-based mediation analysis was performed to examine whether CAR might statistically account for part of the association between Lp(a) and 90-day functional outcome, allowing for exposure–mediator interaction (INT = TRUE). The diagram illustrates the hypothesized relationships among Lp(a), CAR, and outcome, including the indirect pathway through CAR and the direct pathway not through CAR. Effect estimates are presented as the total effect (TE), average direct effect (ADE), and average causal mediation effect (ACME), evaluated at the higher and lower Lp(a) contrast levels (P75 vs P25), with 95% confidence intervals derived from 2,000 bootstrap resamples. All estimates were adjusted for Model 2 covariates. Because Lp(a) and CAR were measured concurrently at baseline, these findings should be interpreted as exploratory rather than as evidence of a definitive causal pathway.

**
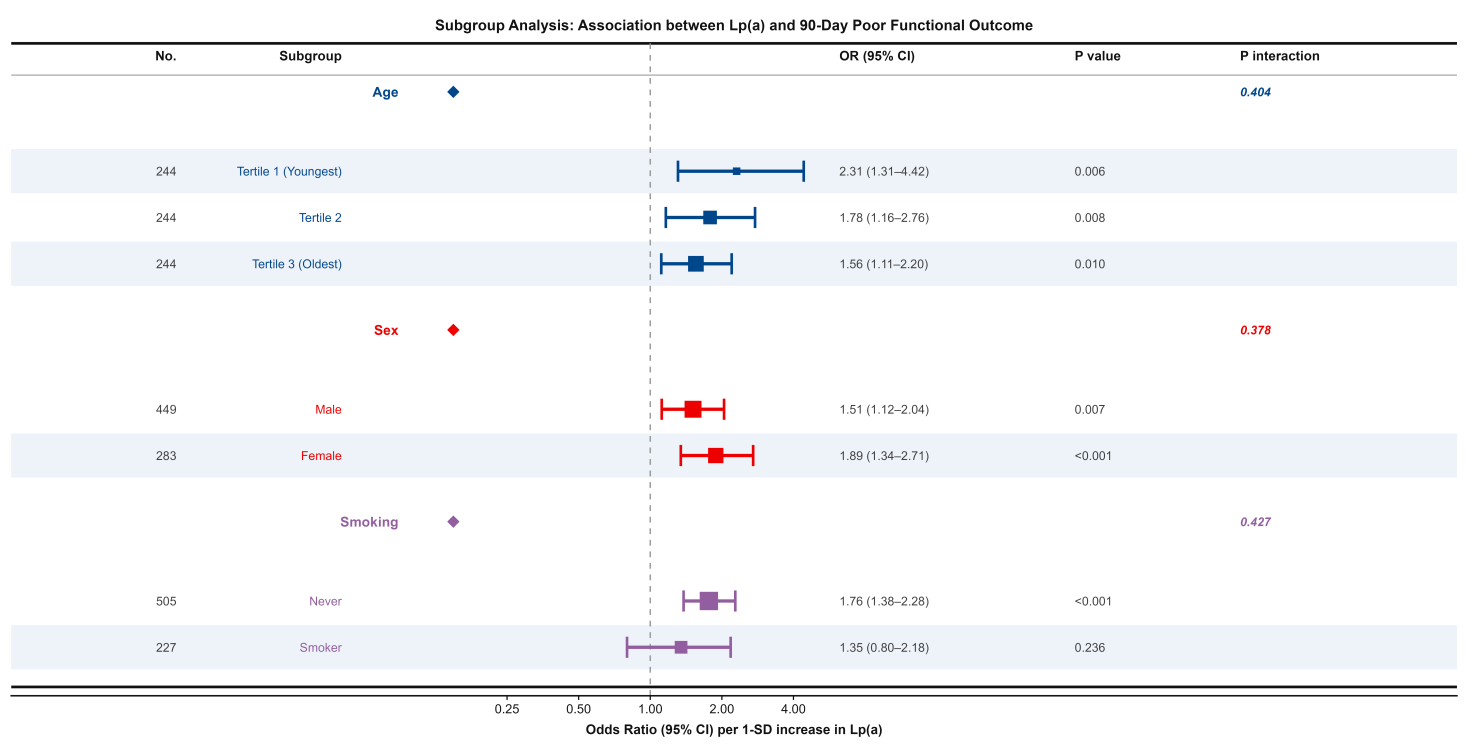
**

**Supplementary Fig. 2.** Subgroup analyses of the association between lipoprotein(a) and 90-day poor functional outcome

Forest plot showing subgroup-specific odds ratios (ORs) and 95% confidence intervals (CIs) for 90-day poor functional outcome per 1–SD increase in lipoprotein(a) [Lp(a)]. Subgroups include age tertiles, sex, and smoking status. P for interaction was calculated by adding the corresponding interaction term between Lp(a) and the subgroup variable in the multivariable logistic regression model. All estimates are adjusted using Model 2 covariates.

**Table S1.** FDR Correction and E-Value Analysis

| Outcome | Exposure | Type | OR (95% CI) | p.value | FDR-adjusted P | E-value |
| --- | --- | --- | --- | --- | --- | --- |
| **14-days Poor Prognosis** | Lpa | Per SD increment | 1.419 (1.174-1.72) | <0.001 | 0.003 | 1.191 |
|  |  | Tertile 3 vs 1 | 2.394 (1.517-3.811) | <0.001 | 0.003 | 1.547 |
|  | SIRI | Per SD increment | 0.925 (0.744-1.088) | 0.381 | 0.761 | 0.962 |
|  |  | Tertile 3 vs 1 | 1.118 (0.707-1.767) | 0.634 | 0.908 | 1.057 |
|  | NLR | Per SD increment | 0.872 (0.631-1.046) | 0.251 | 0.645 | 0.934 |
|  |  | Tertile 3 vs 1 | 1.092 (0.682-1.743) | 0.713 | 0.908 | 1.045 |
|  | MLR | Per SD increment | 1.002 (0.796-1.195) | 0.986 | 0.986 | 1.001 |
|  |  | Tertile 3 vs 1 | 1.18 (0.752-1.851) | 0.472 | 0.839 | 1.086 |
|  | dNLR | Per SD increment | 1.264 (1.031-1.698) | 0.067 | 0.237 | 1.124 |
|  |  | Tertile 3 vs 1 | 1.391 (0.887-2.184) | 0.151 | 0.439 | 1.179 |
|  | CAR | Per SD increment | 1.564 (1.211-2.201) | 0.004 | 0.023 | 1.251 |
|  |  | Tertile 3 vs 1 | 1.684 (1.073-2.656) | 0.024 | 0.096 | 1.298 |
|  | MHR | Per SD increment | 0.92 (0.659-1.15) | 0.567 | 0.908 | 0.959 |
|  |  | Tertile 3 vs 1 | 0.953 (0.602-1.508) | 0.836 | 0.923 | 0.976 |
|  | GLR | Per SD increment | 1.034 (0.856-1.238) | 0.718 | 0.908 | 1.017 |
|  |  | Tertile 3 vs 1 | 1.073 (0.677-1.7) | 0.765 | 0.908 | 1.036 |
| **90-days Poor Prognosis** | Lpa | Per SD increment | 2.115 (1.696-2.659) | <0.001 | <0.001 | 1.454 |
|  |  | Tertile 3 vs 1 | 2.736 (1.519-5.074) | 0.001 | 0.007 | 1.654 |
|  | SIRI | Per SD increment | 0.825 (0.51-1.088) | 0.337 | 0.761 | 0.908 |
|  |  | Tertile 3 vs 1 | 0.962 (0.527-1.757) | 0.899 | 0.928 | 0.981 |
|  | NLR | Per SD increment | 0.746 (0.424-1.072) | 0.262 | 0.645 | 0.864 |
|  |  | Tertile 3 vs 1 | 0.755 (0.412-1.377) | 0.359 | 0.761 | 0.869 |
|  | MLR | Per SD increment | 0.961 (0.687-1.229) | 0.794 | 0.908 | 0.980 |
|  |  | Tertile 3 vs 1 | 0.899 (0.495-1.631) | 0.725 | 0.908 | 0.948 |
|  | dNLR | Per SD increment | 1.157 (0.972-1.396) | 0.083 | 0.266 | 1.076 |
|  |  | Tertile 3 vs 1 | 0.858 (0.485-1.511) | 0.595 | 0.908 | 0.926 |
|  | CAR | Per SD increment | 1.592 (1.278-2.133) | <0.001 | 0.004 | 1.262 |
|  |  | Tertile 3 vs 1 | 2.225 (1.246-4.056) | 0.008 | 0.035 | 1.492 |
|  | MHR | Per SD increment | 1.027 (0.683-1.296) | 0.867 | 0.924 | 1.014 |
|  |  | Tertile 3 vs 1 | 1.289 (0.709-2.352) | 0.404 | 0.761 | 1.136 |
|  | GLR | Per SD increment | 0.944 (0.721-1.202) | 0.659 | 0.908 | 0.972 |
|  |  | Tertile 3 vs 1 | 0.919 (0.52-1.626) | 0.772 | 0.908 | 0.959 |

Abbreviations: OR, odds ratio; CI, confidence interval; SD, standard deviation; FDR, false discovery rate; Lp(a), lipoprotein(a); SIRI, systemic inflammation response index; NLR, neutrophil-to-lymphocyte ratio; MLR, monocyte-to-lymphocyte ratio; dNLR, derived neutrophil-to-lymphocyte ratio; CAR, C-reactive protein-to-albumin ratio; MHR, monocyte-to-high-density lipoprotein ratio; GLR, glucose-to-lymphocyte ratio. All estimates are adjusted using Model 2 covariates.

**Table S2.** Association between inflammatory markers and clinical outcomes using inverse probability of treatment weighting (IPTW) analysis.

| **Outcome** | **Exposure** | **Type** | **Comparison** | **Model** | **OR (95% CI)** | **P value** |
| --- | --- | --- | --- | --- | --- | --- |
| 14-day poor prognosis | CAR | Continuous (per SD) | Per SD increase | Crude | 1.93 (1.03–3.61) | 0.040 |
|  |  |  |  | Model 1 (IPTW) | 1.99 (1.09–3.64) | 0.026 |
|  |  |  |  | Model 2 (IPTW) | 2.19 (1.33–3.59) | 0.002 |
|  |  | Tertiles | T2 vs T1 | Crude | 1.02 (0.69–1.52) | 0.920 |
|  |  |  |  | Model 1 (IPTW) | 0.88 (0.59–1.31) | 0.513 |
|  |  |  |  | Model 2 (IPTW) | 0.86 (0.57–1.29) | 0.459 |
|  |  |  | T3 vs T1 | Crude | 1.86 (1.27–2.72) | 0.001 |
|  |  |  |  | Model 1 (IPTW) | 1.54 (1.05–2.27) | 0.028 |
|  |  |  |  | Model 2 (IPTW) | 1.30 (0.87–1.92) | 0.196 |
|  | GLR | Continuous (per SD) | Per SD increase | Crude | 1.18 (1.01–1.38) | 0.039 |
|  |  |  |  | Model 1 (IPTW) | 1.15 (0.98–1.35) | 0.089 |
|  |  |  |  | Model 2 (IPTW) | 1.04 (0.88–1.22) | 0.637 |
|  |  | Tertiles | T2 vs T1 | Crude | 1.06 (0.72–1.57) | 0.767 |
|  |  |  |  | Model 1 (IPTW) | 0.97 (0.65–1.44) | 0.879 |
|  |  |  |  | Model 2 (IPTW) | 0.88 (0.57–1.33) | 0.535 |
|  |  |  | T3 vs T1 | Crude | 1.53 (1.05–2.23) | 0.029 |
|  |  |  |  | Model 1 (IPTW) | 1.19 (0.80–1.75) | 0.391 |
|  |  |  |  | Model 2 (IPTW) | 1.03 (0.68–1.56) | 0.883 |
|  | Lpa | Continuous (per SD) | Per SD increase | Crude | 1.55 (1.32–1.83) | <0.001 |
|  |  |  |  | Model 1 (IPTW) | 1.51 (1.29–1.78) | <0.001 |
|  |  |  |  | Model 2 (IPTW) | 1.11 (0.94–1.32) | 0.228 |
|  |  | Tertiles | T2 vs T1 | Crude | 1.16 (0.77–1.74) | 0.471 |
|  |  |  |  | Model 1 (IPTW) | 1.20 (0.80–1.82) | 0.379 |
|  |  |  |  | Model 2 (IPTW) | 1.20 (0.79–1.83) | 0.387 |
|  |  |  | T3 vs T1 | Crude | 2.60 (1.77–3.83) | <0.001 |
|  |  |  |  | Model 1 (IPTW) | 2.28 (1.54–3.37) | <0.001 |
|  |  |  |  | Model 2 (IPTW) | 1.73 (1.16–2.58) | 0.007 |
|  | MHR | Continuous (per SD) | Per SD increase | Crude | 0.85 (0.69–1.03) | 0.102 |
|  |  |  |  | Model 1 (IPTW) | 0.90 (0.76–1.07) | 0.233 |
|  |  |  |  | Model 2 (IPTW) | 0.89 (0.74–1.07) | 0.212 |
|  |  | Tertiles | T2 vs T1 | Crude | 0.79 (0.54–1.15) | 0.213 |
|  |  |  |  | Model 1 (IPTW) | 0.89 (0.60–1.31) | 0.549 |
|  |  |  |  | Model 2 (IPTW) | 0.88 (0.59–1.31) | 0.518 |
|  |  |  | T3 vs T1 | Crude | 0.80 (0.55–1.17) | 0.251 |
|  |  |  |  | Model 1 (IPTW) | 1.03 (0.69–1.53) | 0.890 |
|  |  |  |  | Model 2 (IPTW) | 0.90 (0.60–1.34) | 0.596 |
|  | MLR | Continuous (per SD) | Per SD increase | Crude | 1.07 (0.91–1.26) | 0.398 |
|  |  |  |  | Model 1 (IPTW) | 1.05 (0.90–1.21) | 0.556 |
|  |  |  |  | Model 2 (IPTW) | 1.02 (0.89–1.17) | 0.796 |
|  |  | Tertiles | T2 vs T1 | Crude | 0.79 (0.53–1.17) | 0.231 |
|  |  |  |  | Model 1 (IPTW) | 0.73 (0.49–1.08) | 0.115 |
|  |  |  |  | Model 2 (IPTW) | 0.68 (0.45–1.01) | 0.058 |
|  |  |  | T3 vs T1 | Crude | 1.51 (1.04–2.19) | 0.031 |
|  |  |  |  | Model 1 (IPTW) | 1.14 (0.77–1.68) | 0.505 |
|  |  |  |  | Model 2 (IPTW) | 1.08 (0.73–1.59) | 0.708 |
|  | NLR | Continuous (per SD) | Per SD increase | Crude | 1.03 (0.90–1.19) | 0.632 |
|  |  |  |  | Model 1 (IPTW) | 1.05 (0.90–1.22) | 0.554 |
|  |  |  |  | Model 2 (IPTW) | 1.06 (0.90–1.25) | 0.492 |
|  |  | Tertiles | T2 vs T1 | Crude | 1.09 (0.73–1.62) | 0.685 |
|  |  |  |  | Model 1 (IPTW) | 0.99 (0.66–1.50) | 0.979 |
|  |  |  |  | Model 2 (IPTW) | 0.91 (0.59–1.39) | 0.655 |
|  |  |  | T3 vs T1 | Crude | 2.08 (1.42–3.05) | <0.001 |
|  |  |  |  | Model 1 (IPTW) | 1.58 (1.06–2.36) | 0.024 |
|  |  |  |  | Model 2 (IPTW) | 1.06 (0.69–1.61) | 0.798 |
|  | SIRI | Continuous (per SD) | Per SD increase | Crude | 1.06 (0.90–1.25) | 0.478 |
|  |  |  |  | Model 1 (IPTW) | 1.08 (0.90–1.29) | 0.421 |
|  |  |  |  | Model 2 (IPTW) | 1.07 (0.90–1.27) | 0.445 |
|  |  | Tertiles | T2 vs T1 | Crude | 0.96 (0.65–1.43) | 0.841 |
|  |  |  |  | Model 1 (IPTW) | 1.13 (0.75–1.70) | 0.553 |
|  |  |  |  | Model 2 (IPTW) | 1.05 (0.70–1.59) | 0.811 |
|  |  |  | T3 vs T1 | Crude | 1.76 (1.20–2.56) | 0.004 |
|  |  |  |  | Model 1 (IPTW) | 1.55 (1.05–2.30) | 0.028 |
|  |  |  |  | Model 2 (IPTW) | 1.08 (0.72–1.62) | 0.704 |
|  | dNLR | Continuous (per SD) | Per SD increase | Crude | 1.51 (0.68–3.35) | 0.314 |
|  |  |  |  | Model 1 (IPTW) | 1.60 (0.70–3.63) | 0.263 |
|  |  |  |  | Model 2 (IPTW) | 1.52 (0.65–3.57) | 0.334 |
|  |  | Tertiles | T2 vs T1 | Crude | 0.86 (0.57–1.29) | 0.471 |
|  |  |  |  | Model 1 (IPTW) | 0.92 (0.61–1.39) | 0.691 |
|  |  |  |  | Model 2 (IPTW) | 0.93 (0.61–1.43) | 0.741 |
|  |  |  | T3 vs T1 | Crude | 2.24 (1.54–3.27) | <0.001 |
|  |  |  |  | Model 1 (IPTW) | 1.95 (1.32–2.88) | <0.001 |
|  |  |  |  | Model 2 (IPTW) | 1.35 (0.90–2.02) | 0.147 |
| 90-day poor prognosis | CAR | Continuous (per SD) | Per SD increase | Crude | 1.95 (1.08–3.53) | 0.027 |
|  |  |  |  | Model 1 (IPTW) | 2.02 (1.13–3.59) | 0.017 |
|  |  |  |  | Model 2 (IPTW) | 2.04 (1.29–3.24) | 0.002 |
|  |  | Tertiles | T2 vs T1 | Crude | 0.96 (0.55–1.68) | 0.886 |
|  |  |  |  | Model 1 (IPTW) | 0.85 (0.48–1.49) | 0.566 |
|  |  |  |  | Model 2 (IPTW) | 0.90 (0.50–1.62) | 0.733 |
|  |  |  | T3 vs T1 | Crude | 2.14 (1.30–3.52) | 0.003 |
|  |  |  |  | Model 1 (IPTW) | 1.91 (1.15–3.17) | 0.012 |
|  |  |  |  | Model 2 (IPTW) | 1.79 (1.06–3.01) | 0.029 |
|  | GLR | Continuous (per SD) | Per SD increase | Crude | 1.13 (0.95–1.35) | 0.179 |
|  |  |  |  | Model 1 (IPTW) | 1.07 (0.89–1.28) | 0.472 |
|  |  |  |  | Model 2 (IPTW) | 1.03 (0.85–1.25) | 0.766 |
|  |  | Tertiles | T2 vs T1 | Crude | 0.77 (0.45–1.32) | 0.346 |
|  |  |  |  | Model 1 (IPTW) | 0.70 (0.41–1.21) | 0.204 |
|  |  |  |  | Model 2 (IPTW) | 0.66 (0.37–1.18) | 0.162 |
|  |  |  | T3 vs T1 | Crude | 1.35 (0.83–2.19) | 0.223 |
|  |  |  |  | Model 1 (IPTW) | 1.11 (0.68–1.82) | 0.681 |
|  |  |  |  | Model 2 (IPTW) | 0.89 (0.53–1.51) | 0.670 |
|  | Lpa | Continuous (per SD) | Per SD increase | Crude | 2.20 (1.79–2.71) | <0.001 |
|  |  |  |  | Model 1 (IPTW) | 2.14 (1.73–2.64) | <0.001 |
|  |  |  |  | Model 2 (IPTW) | 1.53 (1.24–1.88) | <0.001 |
|  |  | Tertiles | T2 vs T1 | Crude | 1.15 (0.63–2.11) | 0.646 |
|  |  |  |  | Model 1 (IPTW) | 1.28 (0.69–2.37) | 0.429 |
|  |  |  |  | Model 2 (IPTW) | 1.35 (0.73–2.52) | 0.339 |
|  |  |  | T3 vs T1 | Crude | 3.36 (1.99–5.69) | <0.001 |
|  |  |  |  | Model 1 (IPTW) | 3.12 (1.82–5.33) | <0.001 |
|  |  |  |  | Model 2 (IPTW) | 2.37 (1.37–4.08) | 0.002 |
|  | MHR | Continuous (per SD) | Per SD increase | Crude | 0.91 (0.74–1.11) | 0.358 |
|  |  |  |  | Model 1 (IPTW) | 0.97 (0.82–1.15) | 0.719 |
|  |  |  |  | Model 2 (IPTW) | 0.97 (0.82–1.15) | 0.728 |
|  |  | Tertiles | T2 vs T1 | Crude | 0.87 (0.53–1.45) | 0.606 |
|  |  |  |  | Model 1 (IPTW) | 1.04 (0.61–1.77) | 0.881 |
|  |  |  |  | Model 2 (IPTW) | 0.97 (0.57–1.66) | 0.922 |
|  |  |  | T3 vs T1 | Crude | 1.03 (0.63–1.69) | 0.900 |
|  |  |  |  | Model 1 (IPTW) | 1.39 (0.83–2.34) | 0.211 |
|  |  |  |  | Model 2 (IPTW) | 1.19 (0.70–2.03) | 0.511 |
|  | MLR | Continuous (per SD) | Per SD increase | Crude | 1.07 (0.93–1.23) | 0.332 |
|  |  |  |  | Model 1 (IPTW) | 1.05 (0.92–1.21) | 0.442 |
|  |  |  |  | Model 2 (IPTW) | 1.04 (0.90–1.19) | 0.617 |
|  |  | Tertiles | T2 vs T1 | Crude | 1.19 (0.71–2.00) | 0.512 |
|  |  |  |  | Model 1 (IPTW) | 1.13 (0.67–1.91) | 0.653 |
|  |  |  |  | Model 2 (IPTW) | 1.05 (0.62–1.78) | 0.848 |
|  |  |  | T3 vs T1 | Crude | 1.39 (0.84–2.30) | 0.204 |
|  |  |  |  | Model 1 (IPTW) | 1.08 (0.64–1.82) | 0.771 |
|  |  |  |  | Model 2 (IPTW) | 0.98 (0.58–1.66) | 0.949 |
|  | NLR | Continuous (per SD) | Per SD increase | Crude | 1.04 (0.92–1.16) | 0.556 |
|  |  |  |  | Model 1 (IPTW) | 1.04 (0.92–1.17) | 0.519 |
|  |  |  |  | Model 2 (IPTW) | 1.05 (0.93–1.18) | 0.454 |
|  |  | Tertiles | T2 vs T1 | Crude | 0.76 (0.43–1.32) | 0.326 |
|  |  |  |  | Model 1 (IPTW) | 0.69 (0.39–1.22) | 0.198 |
|  |  |  |  | Model 2 (IPTW) | 0.59 (0.33–1.09) | 0.090 |
|  |  |  | T3 vs T1 | Crude | 1.75 (1.08–2.84) | 0.023 |
|  |  |  |  | Model 1 (IPTW) | 1.41 (0.85–2.33) | 0.186 |
|  |  |  |  | Model 2 (IPTW) | 0.83 (0.48–1.42) | 0.489 |
|  | SIRI | Continuous (per SD) | Per SD increase | Crude | 1.03 (0.92–1.15) | 0.589 |
|  |  |  |  | Model 1 (IPTW) | 1.04 (0.92–1.17) | 0.519 |
|  |  |  |  | Model 2 (IPTW) | 1.04 (0.92–1.17) | 0.532 |
|  |  | Tertiles | T2 vs T1 | Crude | 0.96 (0.56–1.66) | 0.890 |
|  |  |  |  | Model 1 (IPTW) | 1.11 (0.64–1.95) | 0.705 |
|  |  |  |  | Model 2 (IPTW) | 1.02 (0.57–1.80) | 0.956 |
|  |  |  | T3 vs T1 | Crude | 1.79 (1.09–2.94) | 0.021 |
|  |  |  |  | Model 1 (IPTW) | 1.65 (0.99–2.76) | 0.057 |
|  |  |  |  | Model 2 (IPTW) | 1.02 (0.60–1.75) | 0.937 |
|  | dNLR | Continuous (per SD) | Per SD increase | Crude | 1.22 (0.96–1.55) | 0.106 |
|  |  |  |  | Model 1 (IPTW) | 1.25 (0.92–1.71) | 0.158 |
|  |  |  |  | Model 2 (IPTW) | 1.24 (0.94–1.62) | 0.124 |
|  |  | Tertiles | T2 vs T1 | Crude | 0.53 (0.30–0.95) | 0.034 |
|  |  |  |  | Model 1 (IPTW) | 0.58 (0.32–1.06) | 0.075 |
|  |  |  |  | Model 2 (IPTW) | 0.57 (0.31–1.06) | 0.077 |
|  |  |  | T3 vs T1 | Crude | 1.66 (1.03–2.65) | 0.036 |
|  |  |  |  | Model 1 (IPTW) | 1.45 (0.89–2.36) | 0.131 |
|  |  |  |  | Model 2 (IPTW) | 0.89 (0.54–1.48) | 0.660 |

Abbreviations: OR, odds ratio; CI, confidence interval; SD, standard deviation; IPTW, inverse probability of treatment weighting; T1/T2/T3, tertile 1/2/3 (T1 as reference). Model 1 (IPTW): weights derived from propensity score models adjusting for age, sex, BMI, smoking, alcohol use, and physical activity. Model 2 (IPTW): weights further adjusted for hypertension, diabetes, heart disease, use of antihypertensive and antidiabetic drugs, TOAST classification, and admission NIHSS. Stabilized weights were used and truncated at the 1st and 99th percentiles. Bold P values indicate statistical significance (P < 0.05).

**Table S3.** Sensitivity analysis of the associations of Lp(a) and inflammatory markers with 14-day and 90-day poor prognosis after re-including patients treated with reperfusion therapy.

| **Exposure** | **Contrast** | **Crude OR (95% CI)** | **P** | **Model 1 OR (95% CI)** | **P** | **Model 2 OR (95% CI)** | **P** |
| --- | --- | --- | --- | --- | --- | --- | --- |
| **14-day poor prognosis** | | | | | | | |
| Lpa | Per 1-SD increase (Z-score) | 1.63 (1.39-1.91) | <0.001 | 1.63 (1.38-1.93) | <0.001 | 1.46 (1.20-1.78) | <0.001 |
|  | Tertile 2 vs 1 | 1.14 (0.76-1.69) | 0.527 | 1.13 (0.74-1.72) | 0.572 | 1.23 (0.78-1.95) | 0.379 |
|  | Tertile 3 vs 1 | 2.56 (1.77-3.75) | <0.001 | 2.43 (1.63-3.65) | <0.001 | 2.23 (1.41-3.55) | <0.001 |
| SIRI | Per 1-SD increase (Z-score) | 1.07 (0.92-1.26) | 0.375 | 1.03 (0.87-1.20) | 0.691 | 0.93 (0.75-1.09) | 0.384 |
|  | Tertile 2 vs 1 | 0.95 (0.64-1.40) | 0.788 | 1.07 (0.71-1.62) | 0.745 | 0.96 (0.61-1.51) | 0.853 |
|  | Tertile 3 vs 1 | 1.85 (1.28-2.69) | 0.001 | 1.67 (1.12-2.50) | 0.013 | 1.12 (0.71-1.77) | 0.619 |
| NLR | Per 1-SD increase (Z-score) | 1.04 (0.89-1.22) | 0.553 | 0.99 (0.82-1.15) | 0.927 | 0.88 (0.64-1.04) | 0.248 |
|  | Tertile 2 vs 1 | 1.03 (0.69-1.52) | 0.899 | 0.90 (0.59-1.36) | 0.616 | 0.88 (0.56-1.38) | 0.575 |
|  | Tertile 3 vs 1 | 2.14 (1.48-3.11) | <0.001 | 1.69 (1.13-2.54) | 0.011 | 1.10 (0.69-1.74) | 0.692 |
| MLR | Per 1-SD increase (Z-score) | 1.07 (0.93-1.26) | 0.334 | 1.05 (0.87-1.22) | 0.574 | 1.01 (0.81-1.20) | 0.939 |
|  | Tertile 2 vs 1 | 0.81 (0.55-1.20) | 0.294 | 0.76 (0.51-1.15) | 0.193 | 0.68 (0.43-1.07) | 0.097 |
|  | Tertile 3 vs 1 | 1.55 (1.08-2.24) | 0.019 | 1.26 (0.85-1.88) | 0.254 | 1.23 (0.79-1.92) | 0.365 |
| dNLR | Per 1-SD increase (Z-score) | 1.54 (1.13-2.30) | 0.024 | 1.48 (1.13-2.16) | 0.024 | 1.24 (1.02-1.63) | 0.072 |
|  | Tertile 2 vs 1 | 0.85 (0.57-1.27) | 0.433 | 0.88 (0.58-1.35) | 0.562 | 0.97 (0.61-1.53) | 0.879 |
|  | Tertile 3 vs 1 | 2.34 (1.62-3.41) | <0.001 | 2.07 (1.40-3.09) | <0.001 | 1.39 (0.89-2.17) | 0.146 |
| CAR | Per 1-SD increase (Z-score) | 2.04 (1.56-2.76) | <0.001 | 2.12 (1.58-2.92) | <0.001 | 1.61 (1.24-2.27) | 0.003 |
|  | Tertile 2 vs 1 | 1.01 (0.68-1.48) | 0.977 | 0.90 (0.59-1.35) | 0.606 | 0.90 (0.56-1.43) | 0.651 |
|  | Tertile 3 vs 1 | 1.86 (1.28-2.70) | 0.001 | 1.72 (1.16-2.57) | 0.008 | 1.69 (1.08-2.65) | 0.022 |
| MHR | Per 1-SD increase (Z-score) | 0.85 (0.64-1.03) | 0.167 | 0.94 (0.72-1.14) | 0.606 | 0.91 (0.66-1.14) | 0.534 |
|  | Tertile 2 vs 1 | 0.76 (0.52-1.10) | 0.140 | 0.91 (0.61-1.35) | 0.629 | 0.87 (0.56-1.35) | 0.539 |
|  | Tertile 3 vs 1 | 0.81 (0.56-1.18) | 0.275 | 1.03 (0.69-1.55) | 0.869 | 0.91 (0.57-1.43) | 0.679 |
| GLR | Per 1-SD increase (Z-score) | 1.20 (1.03-1.39) | 0.017 | 1.14 (0.98-1.34) | 0.095 | 1.04 (0.86-1.24) | 0.675 |
|  | Tertile 2 vs 1 | 1.15 (0.78-1.68) | 0.479 | 1.08 (0.72-1.62) | 0.723 | 1.04 (0.66-1.64) | 0.880 |
|  | Tertile 3 vs 1 | 1.57 (1.08-2.28) | 0.017 | 1.32 (0.89-1.98) | 0.167 | 1.08 (0.69-1.70) | 0.736 |
| **90-day poor prognosis** | | | | | | | |
| Lpa | Per 1-SD increase (Z-score) | 2.42 (2.01-2.94) | <0.001 | 2.54 (2.09-3.12) | <0.001 | 2.23 (1.77-2.82) | <0.001 |
|  | Tertile 2 vs 1 | 1.15 (0.64-2.09) | 0.642 | 1.12 (0.61-2.08) | 0.708 | 1.28 (0.67-2.46) | 0.462 |
|  | Tertile 3 vs 1 | 3.85 (2.35-6.53) | <0.001 | 3.64 (2.18-6.27) | <0.001 | 2.78 (1.54-5.15) | <0.001 |
| SIRI | Per 1-SD increase (Z-score) | 1.04 (0.84-1.22) | 0.637 | 1.01 (0.79-1.20) | 0.878 | 0.83 (0.52-1.09) | 0.343 |
|  | Tertile 2 vs 1 | 1.00 (0.59-1.70) | 0.986 | 1.17 (0.68-2.03) | 0.575 | 0.96 (0.53-1.76) | 0.900 |
|  | Tertile 3 vs 1 | 1.93 (1.20-3.13) | 0.007 | 1.79 (1.09-3.00) | 0.024 | 0.92 (0.51-1.67) | 0.783 |
| NLR | Per 1-SD increase (Z-score) | 1.05 (0.86-1.23) | 0.537 | 1.02 (0.79-1.20) | 0.837 | 0.76 (0.45-1.07) | 0.277 |
|  | Tertile 2 vs 1 | 0.77 (0.45-1.32) | 0.348 | 0.69 (0.39-1.21) | 0.198 | 0.61 (0.33-1.13) | 0.121 |
|  | Tertile 3 vs 1 | 1.93 (1.22-3.09) | 0.006 | 1.54 (0.94-2.55) | 0.086 | 0.78 (0.43-1.40) | 0.401 |
| MLR | Per 1-SD increase (Z-score) | 1.07 (0.88-1.25) | 0.437 | 1.06 (0.83-1.26) | 0.600 | 0.97 (0.70-1.23) | 0.856 |
|  | Tertile 2 vs 1 | 1.22 (0.74-2.00) | 0.440 | 1.20 (0.72-2.01) | 0.488 | 1.06 (0.60-1.90) | 0.832 |
|  | Tertile 3 vs 1 | 1.40 (0.87-2.29) | 0.173 | 1.14 (0.68-1.91) | 0.624 | 0.91 (0.51-1.64) | 0.757 |
| dNLR | Per 1-SD increase (Z-score) | 1.26 (1.06-1.63) | 0.027 | 1.28 (1.07-1.66) | 0.023 | 1.17 (0.98-1.41) | 0.067 |
|  | Tertile 2 vs 1 | 0.46 (0.25-0.81) | 0.009 | 0.47 (0.26-0.85) | 0.014 | 0.50 (0.26-0.95) | 0.037 |
|  | Tertile 3 vs 1 | 1.85 (1.18-2.91) | 0.007 | 1.60 (1.01-2.58) | 0.049 | 0.89 (0.51-1.54) | 0.671 |
| CAR | Per 1-SD increase (Z-score) | 2.16 (1.66-2.87) | <0.001 | 2.24 (1.68-3.04) | <0.001 | 1.66 (1.31-2.25) | <0.001 |
|  | Tertile 2 vs 1 | 1.00 (0.58-1.73) | 0.987 | 0.94 (0.54-1.64) | 0.819 | 0.96 (0.51-1.83) | 0.907 |
|  | Tertile 3 vs 1 | 2.27 (1.42-3.71) | <0.001 | 2.16 (1.32-3.62) | 0.003 | 2.33 (1.31-4.24) | 0.005 |
| MHR | Per 1-SD increase (Z-score) | 0.91 (0.65-1.12) | 0.500 | 1.02 (0.74-1.24) | 0.896 | 1.03 (0.70-1.29) | 0.841 |
|  | Tertile 2 vs 1 | 0.86 (0.53-1.40) | 0.549 | 1.09 (0.65-1.83) | 0.743 | 1.10 (0.62-1.96) | 0.749 |
|  | Tertile 3 vs 1 | 1.03 (0.65-1.66) | 0.889 | 1.38 (0.83-2.30) | 0.217 | 1.20 (0.66-2.18) | 0.556 |
| GLR | Per 1-SD increase (Z-score) | 1.15 (0.96-1.37) | 0.104 | 1.11 (0.91-1.32) | 0.292 | 0.96 (0.74-1.21) | 0.727 |
|  | Tertile 2 vs 1 | 0.94 (0.57-1.56) | 0.811 | 0.85 (0.50-1.44) | 0.549 | 0.72 (0.39-1.33) | 0.300 |
|  | Tertile 3 vs 1 | 1.46 (0.91-2.34) | 0.118 | 1.20 (0.73-1.97) | 0.470 | 0.98 (0.56-1.72) | 0.932 |

Notes: Crude model: unadjusted. Model 1: adjusted for age, sex, body mass index (BMI), smoking status, alcohol consumption, and physical activity. Model 2: further adjusted for hypertension, diabetes, heart disease, use of antihypertensive and antidiabetic drugs, reperfusion therapy status, TOAST classification, and admission NIHSS score. OR, odds ratio; CI, confidence interval. Z-score indicates per 1-SD increase. Tertile comparisons use tertile 1 as the reference group. P-values <0.001 are shown as <0.001.
